# A Meta-analysis of Asbestos and Lung Cancer: Is Better Quality Exposure Assessment Associated with Steeper Slopes of the Exposure–Response Relationships?

**DOI:** 10.1289/ehp.1002879

**Published:** 2011-06-27

**Authors:** Virissa Lenters, Roel Vermeulen, Sies Dogger, Leslie Stayner, Lützen Portengen, Alex Burdorf, Dick Heederik

**Affiliations:** 1 Institute for Risk Assessment Sciences, Division of Environmental Epidemiology, Utrecht University, Utrecht, the Netherlands; 2 Julius Center for Health Studies and Primary Care and Public Health, University Medical Center Utrecht, Utrecht, the Netherlands; 3 Health Council of the Netherlands, the Hague, the Netherlands; 4 Department of Epidemiology and Biostatistics, University of Chicago, Chicago, Illinois, USA; 5 Department of Public Health, Erasmus University Rotterdam, Rotterdam, the Netherlands

**Keywords:** amphiboles, asbestos, chrysotile, lung cancer, meta-analysis

## Abstract

Background: Asbestos is a well-recognized cause of lung cancer, but there is considerable between-study heterogeneity in the slope of the exposure–response relationship.

Objective: We considered the role of quality of the exposure assessment to potentially explain heterogeneity in exposure–response slope estimates.

Data sources: We searched PubMed MEDLINE (1950–2009) for studies with quantitative estimates of cumulative asbestos exposure and lung cancer mortality and identified 19 original epidemiological studies. One was a population-based case–control study, and the others were industry-based cohort studies.

Data extraction: Cumulative exposure categories and corresponding risks were abstracted. Exposure–response slopes [K_L_ (lung cancer potency factor of asbestos)] were calculated using linear relative risk regression models.

Data synthesis: We assessed the quality of five exposure assessment aspects of each study and conducted random effects univariate and multivariate meta-regressions. Heterogeneity in exposure–response relationships was greater than expected by chance (*I*^2^ = 64%). Stratification by exposure assessment characteristics revealed that studies with well-documented exposure assessment, larger contrast in exposure, greater coverage of the exposure history by exposure measurement data, and more complete job histories had higher meta-K_L_ values than did studies without these characteristics. The latter two covariates were most strongly associated with the K_L_ value. Meta-K_L_ values increased when we incrementally restricted analyses to higher-quality studies.

Conclusions: This meta-analysis indicates that studies with higher-quality asbestos exposure assessment yield higher meta-estimates of the lung cancer risk per unit of exposure. Potency differences for predominantly chrysotile versus amphibole asbestos-exposed cohorts become difficult to ascertain when meta-analyses are restricted to studies with fewer exposure assessment limitations.

Asbestos is a potent carcinogen that causes mesothelioma, lung cancer, and laryngeal cancer and may cause ovarian and other cancers (Straif et al. 2009). The current use of asbestos in developing countries is higher than its use in the 1960s in Western Europe and North America (U.S. Geological Survey 2009; Virta 2005). Information about exposure–response relationships is relevant for risk assessment, which is useful for developing preventive strategies. In Western countries, past exposures to asbestos still result in a considerable burden of disease each year (Driscoll et al. 2005; Segura et al. 2003). This burden will remain high in the coming decades, and compensation for those exposed in the past remains an important issue.

There has long been considerable debate about the health risks associated with specific types of asbestos (McDonald and McDonald 1997; Stayner et al. 1996). Mesothelioma occurs more frequently after exposure to amphiboles than after exposure to chrysotile asbestos. The difference in mesothelioma potency, that is, the estimated risk of mesothelioma associated with a unit increase (in fiber-years) in exposure to amphibole versus chrysotile asbestos fibers, is considerable. Recent reviews support this. For example, Hodgson and Darnton (2000) found a potency ratio of 1:100:500 for chrysotile, amosite, and crocidolite, respectively, and Berman and Crump (2008a) reported estimates in the same range. In their most recent analysis, which included more mesothelioma cases from updated cohorts, Hodgson and Darnton (2010) estimated that the ratio of potency for mesothelioma was smaller: 14:1 for amosite versus chrysotile and 54:1 for crocidolite versus chrysotile.

The risk of lung cancer associated with exposure to chrysotile compared with amphibole fibers is still highly contested. Hodgson and Darnton (2000) estimated the potency differential between chrysotile and amphiboles for lung cancer to be between 1:10 and 1:50. Berman and Crump (2008a) reported similar findings—chrysotile was less potent than amphiboles by a factor ranging between 6 and 60, depending on the fiber dimensions considered.

Effect-measure estimates for the relation between lung cancer and asbestos exposure vary strongly between studies. It has been posited that differences in fiber dimension distributions across industries may account for observed differences in potencies (Stanton et al. 1981; Stayner et al. 2008). We hypothesized that differences might be at least partly explained by variation in the quality of the underlying epidemiological data.

Poor quality of exposure estimates may lead to exposure misclassification and underestimation of exposure–response relationships in epidemiological studies (Armstrong 1998). Although quality aspects of epidemiological studies on asbestos have been addressed in earlier reviews (Berman and Crump 2008b; Health Effects Institute 1991; Lash et al. 1997), the influence of quality of the exposure assessment factors on combined effect estimates for asbestos and cancer has not been considered systematically and in a transparent way. This is surprising given the controversy about differences in carcinogenic potency between different types of asbestos and the crucial role of epidemiological evidence in the risk assessment process.

Today, there is more emphasis on the evaluation of study quality of epidemiological studies, specifically the exposure component, in structured reviews and for meta-analyses (Vlaanderen et al. 2008, 2010a, 2010b). We conducted a meta-analysis with specific emphasis on the quality of the exposure assessment used in these studies. We considered whether these quality issues were associated with variability in slopes of the exposure–response relationships.

## Materials and Methods

*Search strategy and inclusion criteria.* We searched the PubMed/MEDLINE (U.S. National Library of Medicine, Bethesda, MD, USA) and EMBASE (Elsevier B.V., Amsterdam, the Netherlands) databases for cohort and case–control studies published before 2010 using combinations of the following key words: amphibole, asbestos, chrysotile, lung cancer, lung neoplasm. The initial search yielded 2,826 articles, of which 1,769 were excluded upon limiting the search to English language and human studies, and we further narrowed the list to 296 upon restriction to cohort and case–control studies. We scrutinized the reference lists of relevant papers for additional publications. A requirement for inclusion in the meta-analysis was that the study should have analyzed the exposure–response relationships quantitatively, with more than one estimate of cumulative exposure (CE), and with excess risk expressed per fiber-years per milliliter or risk per million particles per cubic foot year. A brief note on these two exposure assessment methods: Before the 1970s, airborne asbestos levels were usually measured with the midget impinger method by trapping total airborne (including nonfibrous) dust particles and counting via light microscope (Gibbs 1994). Membrane filter-based methods replaced the midget impinger method; fibers, generally defined as structures more than 5 μm in length with an aspect ratio ≥ 3:1, are identified and counted via phase-contrast microscopy (PCM) or, more recently, with transmission electron microscopy (TEM).

Studies on nonoccupational exposures were not considered eligible. Recent studies that made use of qualitative or semiquantitative exposure assessment approaches were not included in the evaluation. This yielded 19 cohort studies; 18 industry-based studies, including one nested case–control study, and one population-based case–control study [Table 1; see also Supplemental Material, Table 1 (http://dx.doi.org/10.1289/ehp.1002879)].

**Table 1:** Cohort and case–control studies included in the meta-analysis.

Study no.	Cohort	Primary reference	*n*	Fiber type*a*	CF*b*	Ratio highest: lowest CE midpoints	Exposure duration	Measurement coverage*c*
1		Quebec, Canada, mines and mills		Liddell et al. 1997		~ 11,000		Chry		I		1,000		~ 1904–1976		~ 25%
2		Italy, mine and mill (Balangero)		Pira et al. 2009		1,056		Chry		NA		13		1916–1990		24%
3		Connecticut, friction products plant		McDonald et al. 1984		3,513		Chry		E		27		1913–1977		~ 30%
4		South Carolina, textile plant		Hein et al. 2007		3,072		Chry		I		267		1896–1977		> 58%
5		North Carolina, textile plants		Loomis et al. 2009		5,770		Chry		I		1,066		1925–1994*d*		> 74%
6		Wittenoom, Australia, mine and mill		Berry et al. 2004		6,358		Croc		NA		1,999		1937–1966		< 5%
7		Paterson, NJ, insulation factory		Seidman et al. 1986		820		Am		NA		139		1941–1954		0%
8		Tyler, TX, insulation factory		Levin et al. 1998		1,121		Am		NA		33		1954–1972		~ 25%
9		Libby, MT, vermiculite mines and mills		Sullivan 2007		1,672		Tre		I		74		1935–1990		47%
10		UK, friction products factory (Ferodo)		Berry and Newhouse 1983		13,460		Mix		NA		51		1910–1979		19%
11		Ontario, Canada, cement factory		Finkelstein 1984		740		Mix		E		17		1948–1977		> 80%
12		New Orleans, LA, cement plants*e*		Hughes et al. 1987		6,931		Mix		I		61		1937–1972		61%
13		Sweden, cement plant		Albin et al. 1990		2,898		Mix		I		28		1907–1977		30%
14		Belgium, cement plant		Lacquet et al. 1980		1,973		Mix		NA		80		1928–1977		12%
15		USA, factory retirees (Johns Manville)		Enterline et al. 1987		1,074		Mix		E		16		1890–1980		~ 30%
16		USA and Canada, insulation workers		Selikoff and Seidman 1991		17,800		Mix		E		10		~ 1920–1986		0%
17		Pennsylvania, textile plant		McDonald et al. 1983		4,024		Mix		E		22		~ 1900–1967		~ 55%
18		Rochdale, UK, textile plant		Peto et al. 1985		3,211		Mix		I		43		1933–1978		60%
19		Stockholm County, Sweden, population-based case–control study		Gustavsson et al. 2002		1,038 cases, 2,359 referents		Mix		NA		> 100		~ 1925–1974		~ 10%
**a**Predominant fiber type: Am, amosite; Chry, chrysotile; Croc, crocidolite; Mix, mixed; Tre, tremolite. **b**Conversion factor (CF) indicates whether measurements of particles (million particles per cubic foot) were converted to fibers per milliliter with an internally (I) or externally (E) derived conversion factor based on paired measurements or a generic factor, respectively. NA (not applicable) indicates that a conversion factor was not applied because exposures were expressed in units of fiber-years per milliliter. **c**Measurement coverage indicates the proportion of the exposure history that was covered by exposure measurements (impinger or PCM based). **d**Textile operations began before 1925, and one plant ceased production after 1994. **e**Results for Hughes et al. (1987), originally stratified by fiber type, were combined for this meta-analysis.																

*Data extraction and calculation of exposure–response slopes.* Key study characteristics, including descriptors of the exposure assessment, were extracted. For each of these studies, different measures of association were reported, most commonly standardized mortality ratios and in some cases relative risks (RRs) or odds ratios. Although there are fundamental differences between these types of risk measures, for the purpose of this meta-analysis, all study designs were included and all measures of association were considered estimates of the RR.

The outcome analyzed in the meta-analysis was the slope of the exposure–response relation, often referred to as the “lung cancer potency factor” and denoted by K_L_ in the asbestos literature. We used the widely applied U.S. Environmental Protection Agency (1986) linear RR model, RR = α(1 + K_L_ × CE), where α is an intercept parameter representing the background rate of lung cancer and K_L_ is the slope of increase in the RR per unit of CE to asbestos (in fiber-years per milliliter). If available, we used associations for 10-year lagged CE (six studies) to account for latency (U.S. Environmental Protection Agency 1986); otherwise, we used associations with unlagged CE (13 studies). The values for K_L_ were obtained by fitting Poisson regression models with PROC NLMIXED (version 9.2; SAS Institute Inc., Cary, NC, USA). In our primary analyses, we did not restrict the intercept (α) in the calculation of the exposure–response curve. Data extracted from each study to derive K_L_ values are provided in Supplemental Material, Appendix 1 (http://dx.doi.org/10.1289/ehp.1002879). Gustavsson et al. (2002) presented the risk estimate for their study per unit of exposure, and we derived the K_L_ for their study using the formula provided in the paper for the RR at a cumulative dose of *x* fiber-years: RR = 1.494^ln(^*^x^*^+1)^.

The predominant fiber type each study population was exposed to was ascertained from the literature. Some populations were exposed to both chrysotile and amphiboles (including amosite, crocidolite, and tremolite) and were categorized as having experienced “mixed” exposure. Assigned fiber type was in agreement with previous reviews by Berman and Crump (2008a, 2008b) and Hodgson and Darnton (2000).

*Quality of exposure assessment.* Several publications describe generic frameworks to assess the quality of human observational studies for risk assessment (Goldbohm et al. 2006; Stroup et al. 2000; Swaen 2006; Vlaanderen et al. 2008; World Health Organization 2000). From these publications and the asbestos literature, we classified asbestos exposure assessment for each study according to the following characteristics.

Documentation. Whether authors sufficiently described the exposure assessment in terms of number of dust or fiber measurements, variability in exposure within and between exposure categories, details about analytical procedures, and so forth.

CE ratio. As an indication of contrast in exposure within a cohort study, for each study, we estimated the ratio between the average values of exposure within the highest versus lowest CE categories. If the average was not reported, we derived the ratio of the estimated midpoint of each category, with the midpoint of an unbounded upper CE category calculated as its lower bound multiplied by 5/3 (Berman and Crump 2003). Under most circumstances, a limited contrast in exposure intensity increases the likelihood of an attenuated exposure–response association due to an unfavorable partitioning of the variance within and between exposure categories (Tielemans et al. 1998). Hygiene strategies are therefore aimed at maximizing the contrast by choosing the optimal job title structure or estimating exposure on the basis of more detailed exposure determinants than job title alone (Kromhout and Heederik 1995; Loomis and Kromhout 2004). When differences in exposure between categories are small, exposure misclassification is likely to be relatively influential. The median CE ratio of all 19 studies was 51; thus, we classified studies with ratios < 50 as less informative with regard to the contrast in exposure.

Conversion factor. Studies were classified according to the use of internal or external measurement conversion factors to account for changes over time in analytical and measurement techniques. Results obtained by older dust measurement techniques based on impinger sampling (million particles per cubic foot) can be crudely converted into results that would be obtained using PCM and fiber counting (concentrations of fibers > 5 μm in length per milliliter of air) available since the 1960s. Such conversions were necessary in the context of epidemiological studies in which the population was followed over a long period of time (e.g., > 30 years). Conversion factors may differ for different environments (Dement et al. 2009). In some cohort studies, comparison studies were conducted to derive internal conversion factors for various subgroups of a cohort (e.g., Hein et al. 2007). In others, external conversion factors were used that were obtained from studies other than the cohort under study (e.g., Liddell et al. 1997). The use of external conversion factors may result in increased imprecision and exposure misclassification for the cohort compared with the use of internal conversion factors (National Research Council 1984). For the cohorts for which only PCM-based exposure estimates were reported (in fiber-years per milliliter), no conversion factor was required, and these studies were classified as NA (not applicable) and grouped with the “internal conversion factor” studies.

Coverage of exposure data. Percentage of the accumulated work history years temporally covered by exposure measurement data provides an indication of the extent temporal back-extrapolation or reconstruction approaches were used in the exposure estimation process (Vlaanderen et al. 2010a). An optimal evaluation method would estimate the coverage using person-time information of the cohort. Because this was not available for each cohort, a crude estimate was made on the basis of cohort entry times, exposure duration, and the end of the exposure period.

Job histories. We considered whether the job history information was sufficiently complete and detailed to capture changes in job titles or tasks over time and between companies, and sufficiently refined or appropriately used in a way that captured differences in exposure between jobs.

Three independent reviewers (D.H., A.B., V.L.) performed the quality assessment. When evaluations differed, scores were discussed and a consensus score was assigned.

*Statistical analyses.* Meta-analysis and meta-regression models were fitted using restricted maximum likelihood (REML) estimation with SAS PROC MIXED (Thompson and Sharp 1999; van Houwelingen et al. 2002). The input data were K_L_ values and variances of the K_L_ values for each study. The *I*^2^ statistic was calculated to quantify the percentage of total variation across studies attributable to heterogeneity rather than to chance (Higgins and Thompson 2002). We applied random effects meta-analysis for the primary analyses because there was evidence of heterogeneity; specifically, *I*^2^ > 50% (Higgins et al. 2003). We conducted subgroup analyses or univariate meta-regression of categorical study-level descriptors of the exposure assessment strategy to explore whether the *a priori* specified covariates explained any between-study heterogeneity in K_L_. The five covariates were *a*) exposure documentation (insufficient or sufficient), *b*) CE ratio [≤ 50 or > 50 for the ratio of the average (or midpoint) value of the highest vs. lowest exposure category], *c*) type of measurement conversion factor (external vs. internal or NA), *d*) coverage of exposure history by measurement data (≤ 30% vs. > 30%), and *e*) sufficiency of job histories (insufficient or sufficient). We estimated meta-K_L_ values according to fiber type and strata of the five covariates and tested for heterogeneity between strata with a (type 3) *F*-test. A *p*-value of < 0.10 was considered statistically significant. Fiber type was categorized into chrysotile, amphiboles, mixed, and amphiboles plus mixed exposure.

We used multivariate random effects meta-regression to investigate how the categorical covariates affected meta-K_L_ values (Thompson and Sharp 1999; van Houwelingen et al. 2002). Fiber type was dichotomized into chrysotile versus amphiboles/mixed exposure to minimize the degrees of freedom and thus preserve statistical power, and because the difference between potencies for (close to pure) chrysotile exposure versus exposure involving amphiboles was one of the primary research questions. Study-specific K_L_ values were modeled as the dependent variable, and fiber type and individual covariates (descriptors of exposure assessment) were included as the independent variables in univariate, bivariate, and multivariate meta-regression models. We compared model fit with Akaike’s information criterion (AIC) and examined *p*-values of (type 3) *F*-tests. We also evaluated models including interaction terms for fiber type × covariate (*p*-value < 0.05 considered significant). To complement the meta-regression analyses involving all 19 studies, we stepwise excluded studies based on exposure assessment quality aspects and explored the effect of this strategy on the meta-K_L_ value and intercept (α).

To explore the influence of the selected exposure–response model on results, we performed sensitivity analyses using different study-specific K_L_ values. For many studies (*n* = 11), the midpoint of the highest exposure category had to be estimated because an unbounded upper range was reported, and this point could be highly influential with regard to the K_L_ slope. Therefore, we recalculated the K_L_ values excluding the highest exposure category. We also derived K_L_ values from a model with a fixed intercept (α = 1). Others have allowed the intercept (α) of the exposure–response curve to depart from a fixed value of 1 (= RR at zero exposure) to allow for confounding, most likely by smoking or the healthy worker effect (Berman and Crump 2008b; U.S. Environmental Protection Agency 1986), or truncated K_L_ to zero to avoid negative exposure–responses slopes and allowed a maximal slope value of 2 (Berman and Crump 2008b). We therefore repeated analyses using effect-measure estimates from two previous meta-analyses, Berman and Crump (2008a, 2008b) and Hodgson and Darnton (2000). Berman and Crump (2008a, 2008b) also presented K_L_ values. Hodgson and Darnton (2000) used an average CE for each cohort to derive a percentage excess lung cancer risk per unit of CE (R_L_), which represents a “cohort average” potency estimate rather than an internal exposure–response estimate for each study, as with the K_L_. Finally, we performed an exclusion sensitivity analysis to evaluate the effect on the meta-K_L_ of excluding studies one at a time. Too few studies reported effect-measure estimates for different exposure lags, so it was not possible to do a sensitivity analysis using K_L_ values calculated with lagged and unlagged CEs.

Publication bias was assessed by constructing a funnel plot of precision (SE) versus K_L_ values, supplemented by Egger’s linear regression test for funnel plot asymmetry (Egger et al. 1997).

## Results

**Figure 1:**
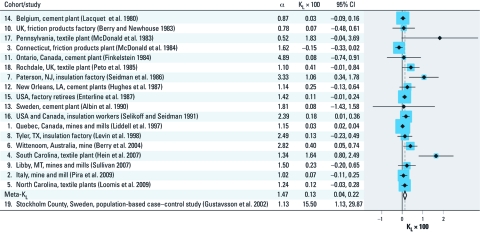
Forest plot with K_L_ × 100 values (closed diamonds) and 95% CIs (lines) of included studies (for study numbers and details, see Table 1). The size of each square is proportional to the weight the study received in the meta-analysis. The open diamond denotes the meta-K_L_. Intercepts (α), K_L_ values, and 95% CIs are listed. For scaling purposes, study 19 (Gustavsson et al. 2002) is not displayed in the plot.

Table 1 summarizes the characteristics of included studies, including fiber type [see also Supplemental Material, Table 1 and Appendix (http://dx.doi.org/10.1289/ehp.1002879)]. The estimated study-specific K_L_ values varied by several orders of magnitude (Figure 1). The intercepts (α) varied less strongly, but the difference between the highest and lowest intercept was still a factor of 9. The funnel plot of the cohort studies (Supplemental Material, Figure 1) provided some evidence of potential publication bias, and this was corroborated by Egger’s regression test (bias 0.71; *p* = 0.03). We found a high degree of heterogeneity between studies (*I*^2^ = 64%). Under a random effects model, we found a meta-K_L_ (× 100) of 0.13 [95% confidence interval (CI): 0.04, 0.22] with an intercept of 1.47. This represents a RR of 1.66 (95% CI: 1.53, 1.79) for lung cancer for each 100 fiber-years/mL increase in exposure based on the aforementioned model RR = α(1 + K_L_ × CE), or 1.66 = 1.47(1 + 0.13). The case–control by Gustavsson et al. (2002) had a remarkably high K_L_ value, one to three orders of magnitude higher than the other studies: 15.50 versus –0.15 to 1.83 (Figure 1). However, a sensitivity analysis showed that the influence of this study on the overall K_L_ value was negligible, consistent with expectations given its large standard error (Table 2).

**Table 2:** Univariate associations between K_L_ factors stratified on fiber type and different characteristics of exposure assessment.

Inclusion	No. of studies	*I*^2 ^(%)	Meta-K_L_ × 100 (95% CI)	*p*-Value*a*	AIC	Studies included*b*
All studies		19		64.1		0.13 (0.04, 0.22)		–		28.2		1–19
All but Gustavsson et al. 2002		18		62.7		0.13 (0.04, 0.22)		–		18.0		1–18
Fiber												
Chrysotile*c*		5		79.8		0.04 (–0.05, 0.12)				28.6		1–5
Amphiboles (crocidolite, amosite, tremolite)		4		45.5		0.33 (0.09, 0.56)		0.06*d*				6–9
Mixed		10		26.4		0.13 (0.03, 0.23)						10–19
Amphiboles and mixed		14		38.7		0.18 (0.07, 0.29)		0.10*e*		28.7		6–19
Documentation												
Insufficient*c*		8		62.7		0.11 (–0.04, 0.26)		0.46		30.6		2, 3, 6, 7, 10, 11, 14, 16
Sufficient		11		67.4		0.18 (0.04, 0.33)						1, 4, 5, 8, 9, 12, 13, 15, 17–19
CE ratio (highest:lowest exposure category)												
≤ 50*c*		9		43.7		0.10 (–0.05, 0.26)		0.38		30.3		2, 3, 8, 11, 13, 15, 16, 17, 18
> 50		10		73.7		0.20 (0.04, 0.35)						1, 4, 5, 6, 7, 9, 10, 12, 14, 19
Conversion factor (million particles per cubic foot to fiber-years/mL)												
External or never PCM*c*		6		73.4		0.12 (–0.07, 0.30)		0.69		30.8		3, 7, 11, 15, 16, 17
Internal or always PCM		13		59.9		0.16 (0.03, 0.28)						1, 2, 4, 5, 6, 8–10, 12–14, 18, 19
Coverage of exposure data												
≤ 30%*c*		12		57.7		0.08 (–0.01, 0.18)		0.08		27.6		1, 2, 3, 6, 7, 8, 10, 13, 14, 15, 16, 19
> 30%		7		63.3		0.27 (0.08, 0.46)						4, 5, 9, 11, 12, 17, 18
Job histories												
Insufficient*c*		6		26.8		0.03 (–0.10, 0.17)		0.08		27.9		1, 3, 5, 11, 12, 13
Sufficient		13		64.4		0.19 (0.08, 0.30)						2, 4, 6, 7, 8, 9, 10, 14–19
**a**Difference between subgroups (*F*-test). **b**Study numbers are given in Table 1. **c**Reference category in meta-regression analyses. **d**Test for difference between meta-K_L_ values for chrysotile, amphiboles, and mixed strata. **e**Test for difference between meta-K_L_ values for chrysotile versus the amphiboles and mixed strata.												

*Scoring of exposure assessment.* Generally, documentation of the exposure assessment component of most studies was not informative and often not sufficiently transparent. Information available from many of the original papers was extremely limited, sometimes consisting of only a few sentences. In contrast, for some studies the exposure assessment component is described in detail in separate publications (Dement et al. 1982, 1983; Gibbs 1994; Gibbs and Lachance 1972). Often only average concentrations were given, without details about the number of measurements, variability in exposure within and between workers or job title categories, or details on the exposure assessment methodology (analytical technique, measurement protocol, or sampling strategy). We scored individual studies (indicated by study number as defined in Table 1) as follows:

Documentation: studies with an insufficiently documented exposure assessment, including studies that made use of external exposure data and exposure assessment strategies that were not considered sufficiently accurate (2, 3, 6, 7, 10, 11, 14, 16)
CE ratio: studies with a factor of < 50 in contrast between the highest and the lowest CE categories (2, 3, 8, 11, 13, 15–18)
Conversion factor: studies with undocumented or external conversion factors to convert dust measurements in million particles per cubic foot (or particles per milliliter) to fiber concentrations (3, 7, 11, 15, 16, 17)
Coverage of exposure data: studies with ≤ 30% of the exposure history covered by impinger or PCM-based measurements (1, 2, 3, 6, 7, 8, 10, 13–16, 19)
Job histories: studies with documented problems with the sufficiency or accuracy of the job history information (1, 3, 5, 11, 12, 13).

*Univariate meta-regression.* We observed considerable differences in meta-K_L_ values stratified by fiber type when we considered all studies. The meta-K_L_ value was roughly eight times higher for exposure to amphiboles versus chrysotile fibers (Table 2). The difference between the meta-K_L_ for amphiboles (0.33; 95% CI: 0.09, 0.56; four studies) and for chrysotile (0.04; 95% CI: –0.05, 0.12; five studies) became slightly less pronounced when we excluded the Quebec mine study (chrysotile meta-K_L_ = 0.07; 95% CI: –0.10, 0.25). Studies with mixed fiber exposure (*n* = 10) had an intermediate meta-K_L_ value (0.13; 95% CI: 0.03, 0.23) that was approximately three times higher than the estimated meta-K_L_ for chrysotile alone (Table 2).

In general, we observed that *a priori* identified aspects of the exposure assessment strategy were individually associated with K_L_ values (univariate estimates; Table 3) and that studies with better exposure assessment characteristics had higher K_L_ values in the stratified analysis (Table 2). These differences in stratum-specific K_L_ values were most pronounced for studies with greater coverage compared with studies with limited coverage of exposure history by exposure measurement data, and for studies with sufficient compared with insufficient job history information (Table 2).

**Table 3:** Univariate and bivariate meta-regression models of K_L_, with fiber type and exposure assessment covariates modeled as independent variables.

Model	β-Coefficient (95% CI)	*p*-Value	AIC
Univariate						
Fiber (amphiboles/mixed)		0.13 (–0.03, 0.29)		0.10		28.7
Documentation (sufficient)		0.07 (–0.13, 0.28)		0.46		30.6
CE ratio (> 50)		0.09 (–0.13, 0.31)		0.38		30.3
Conversion factor (internal)		0.04 (–0.18, 0.26)		0.70		30.8
Coverage of exposure data (> 30%)		0.19 (–0.02, 0.40)		0.08		27.6
Job histories (sufficient)		0.16 (–0.02, 0.33)		0.08		27.9
Bivariate						
Fiber (amphiboles/mixed)		0.14 (–0.03, 0.32)		0.09		30.9
Documentation (sufficient)		0.08 (–0.09, 0.25)		0.34		
Fiber (amphiboles/mixed)		0.15 (–0.04, 0.34)		0.12		30.9
CE ratio (> 50)		0.09 (–0.10, 0.28)		0.33		
Fiber (amphiboles/mixed)		0.15 (–0.02, 0.32)		0.08		31.0
Conversion factor (internal)		0.07 (–0.11, 0.26)		0.40		
Fiber (amphiboles/mixed)		0.13 (0.00, 0.26)		0.05		27.1
Coverage of exposure data (> 30%)		0.18 (0.01, 0.36)		0.04		
Fiber (amphiboles/mixed)		0.05 (–0.22, 0.31)		0.71		30.1
Job histories (sufficient)		0.13 (–0.14, 0.40)		0.31		
Exposures to amphiboles and mixed fiber types were grouped. For each covariate (fiber type and five exposure assessment covariates), a reference category was chosen as denoted in Table 2.						

*Multivariate meta-regression.* Exposure assessment covariates were positively associated with the meta-K_L_ when included with fiber type in one model (bivariate estimates; Table 3). As in the univariate analyses, coverage of exposure data and sufficiency of job histories also had a relatively strong influence on the K_L_ value. The estimated effect of fiber type was similar after adjustment for all study characteristics except job history, which reduced the K_L_ for amphiboles/mixed versus chrysotile from 0.13 (95% CI: –0.03, 0.29) to 0.05 (95% CI: –0.22, 0.31). However, the strong correlation between job history and fiber type (as evident from the wide CIs) complicates inferences from this model. Adjusting for coverage of exposure data did not affect K_L_ value for fiber type. Interactions between fiber and covariates were not significant (*p* ≥ 0.05; data not shown).

Inclusion of various combinations of two covariates, in addition to fiber type, revealed that coverage of exposure data and sufficiency of job histories produced the best-fitting model, with a slightly lower AIC than univariate, fiber plus one covariate, or multivariate (all covariates included) models (data not shown).

*Exclusion of poorer-quality studies.* We found a clear trend of increasing K_L_ values when we incrementally excluded poorer-quality studies on the basis of exposure assessment criteria (Table 4). We first excluded all studies without sufficient documentation (eight studies). We subsequently excluded studies with external conversion factors (two additional studies), and then studies with documented problems with the job histories (four additional studies). Lastly, we excluded studies with a lower contrast in exposure (CE ratio ≤ 50; two additional studies) and ≤ 30% coverage of the exposure history by exposure measurement data (one excluded, two remaining). The meta-K_L_ increased from 0.13 (95% CI: 0.04, 0.22) to 0.55 (95% CI: 0.11, 0.99). When we changed the order in which we excluded studies to exclusion based on low coverage of exposure data, then based on sufficiency of job histories, and then lastly based on external conversion factors, we still observed a similar trend of increasing K_L_ values with the application of an increasing number of quality criteria (data not shown). We also applied an alternative exclusion strategy, in which we investigated the association between the K_L_ values and the number of unmet exposure assessment criteria. This sensitivity analysis was limited in the sense that studies with the same number of weaknesses may suffer from different weaknesses, which might be difficult to compare directly. However, this analysis also yielded higher meta-K_L_ for studies with fewer limitations in exposure assessment [see Supplemental Material, Table 2 (http://dx.doi.org/10.1289/ehp.1002879)].

**Table 4:** Results from the random effects meta-analysis in which studies were excluded stepwise with specific exposure assessment descriptors.

Incremental exclusion	No. of studies included	*I*^2 ^(%)	Meta-α (95% CI)	Meta-K_L_ × 100 (95% CI)	AIC	Studies included*a*
None (all 19 studies)		19		64.1		1.47 (1.14, 1.81)		0.13 (0.04, 0.22)		28.2		1–19
Studies with insufficient documentation		11		67.4		1.29 (0.87, 1.71)		0.18 (0.04, 0.33)		30.6		1, 4, 5, 8, 9, 12, 13, 15, 17–19
Studies with external conversion factors		9		68.5		1.38 (0.89, 1.86)		0.19 (0.03, 0.35)		30.6		1, 4, 5, 8, 9, 12, 13, 18, 19
Studies with insufficient job histories		5		73.7		1.44 (0.78, 2.10)		0.36 (0.10, 0.61)		26.4		4, 8, 9, 18, 19
Studies with CE ratio ≤ 50		3		84.4		1.32 (0.50, 2.14)		0.56 (0.12, 1.00)		25.0		4, 9, 19
Studies with coverage ≤ 30%		2		88.4		1.42 (0.40, 2.44)		0.55 (0.11, 0.99)		25.3		4, 9
**a**Study numbers are given in Table 1.												

*Sensitivity analyses.* We also observed the trend shown in Table 4 of increasing potency values for studies with fewer limitations when we used study-specific K_L_ values derived by Berman and Crump (2008b) or R_L_ values derived by Hodgson and Darnton (2000) [see Supplemental Material, Table 3 (http://dx.doi.org/10.1289/ehp.1002879)], with increases from 0.06 to 0.57 for meta-K_L_ × 100 and from 1.69 to 5.35 for meta-R_L_ based on all studies versus the highest-quality studies, respectively (see Supplemental Material, Table 4). This trend of increasing meta-K_L_ values was less consistent but generally apparent for the analyses in which we used K_L_ values that had been calculated omitting the upper CE category or with the regression line forced through an intercept of 1 (see Supplemental Material, Table 5). The pattern for meta-K_L_ values stratified by exposure quality determinants was comparable in the sensitivity analyses, with markedly higher meta-K_L_ values for > 30% versus ≤ 30% coverage of exposure history by exposure measurement data, and for sufficient versus insufficient job history information, for all scenarios except for the K_L_ values calculated with an intercept fixed to 1 (see Supplemental Material, Tables 6 and 7). The exclusion sensitivity plot revealed that excluding the Quebec mine study or the South Carolina textile plant study had the greatest influence on the meta-K_L_, leading to the greatest increase and decrease in the meta-K_L_, respectively [see Supplemental Material, Figure 2 (http://dx.doi.org/10.1289/ehp.1002879)].

## Discussion

Potency differences between chrysotile and amphiboles have received much attention in risk assessments for asbestos and may explain heterogeneity in exposure–response estimates between studies (Berman and Crump 2008a; Hodgson and Darnton 2000; Mossman et al. 1990; Nicholson 1991). We observed that variables that described aspects of the exposure assessment strategy were individually associated with variability in lung cancer potency factors (K_L_ values). For instance, coverage of exposure history by exposure measurement data and sufficiency of job histories were associated with the K_L_ values. Contrast in exposure, expressed as the ratio between the average or midpoint values of the highest and lowest CE categories for individual studies, was also positively associated with the K_L_ values. If we incrementally excluded studies that did not satisfy quality of the exposure assessment criteria, we observed a gradual increase in the meta-K_L_ values. The increase in K_L_ values was not explained by the relatively high K_L_ value for the general population-based case–control study (Gustavsson et al. 2002). Moreover, meta-regression analyses revealed that individual exposure assessment covariates were positively associated with K_L_ values, even upon adjustment for fiber type. However, estimated lung cancer potency differences between amphiboles and chrysotile decreased after adjustment for sufficiency of job history information, although interpretation of bivariate meta-regression results was hindered by potential multicollinearity between fiber type and exposure assessment covariates. Analyses presented in this article cast doubt on the conclusion that the epidemiological evidence for lung cancer strongly supports a difference in potency for different fiber types.

Our findings detract from the amphibole hypothesis, whose proponents argue that the carcinogenicity of chrysotile is due to contamination by amphiboles (Mossman et al. 1990; Stayner et al. 1996): first, there are too few studies with exposure assessment of sufficient quality to adequately address this question of potency; second, the meta-K_L_ values are highly sensitive to exposure assessment covariates.

We observed the overall pattern in these results with differently derived K_L_ values: exposure–response curves fitted with an unrestricted intercept or with a fixed intercept (α = 1), and with the uppermost CE category excluded. Interestingly, we also observed similar patterns with K_L_ values and excess risk (R_L_) values extracted from reviews by Berman and Crump (2008a, 2008b) and Hodgson and Darnton (2000), indicating that the observed pattern of associations between asbestos potency and covariates that describe the exposure assessment of studies is robust and not dependent on the method by which the potency factors are derived. A variable intercept model allowed us to more accurately assess the influence of exposure assessment aspects on heterogeneity and exposure–response slopes. For regulatory processes, risk assessors may calculate exposure–response relationships by forcing regression lines through an intercept of 1. In this meta-analysis fixed-intercept meta-K_L_ values were steeper because, for most studies, the unrestricted intercept of the exposure–response was larger than RR = 1, possibly reflecting combined confounding bias (i.e., due to smoking) and exposure misclassification bias. Although the pattern of change in lung cancer potency factors was generally robust in sensitivity analyses comparing potency factors included by two aforementioned recent risk assessments, and variable- and fixed-intercept models, the absolute meta-potency values were sensitive to model assumptions.

The pattern observed, with higher K_L_ values for studies with fewer limitations in the exposure assessment component, is in agreement with epidemiological theory on information bias and exposure misclassification (Armstrong 1998). Examples from the literature show that in most cases random error or nondifferential misclassification results in underestimation of the slope of exposure–response relationship, in combination with a loss of power and potential changes to the shape of the exposure–response relationship (Armstrong 1998; Heederik and Attfield 2000). Although in some scenarios nondifferential exposure misclassification may lead to an overestimation of the slope of exposure–response curves (Dosemeci et al. 1990; Loomis and Kromhout 2004), it is generally accepted that underestimation is most likely to occur. Exposure categorization, when done in an optimal way, is usually associated with less underestimation but a more considerable reduction of the power of a study to detect an association because the error has a Berkson error structure (Armstrong 1998; Tielemans et al. 1998). Categorization has been applied in all asbestos cohort studies. However, CE is calculated on the basis of different sources of information, including information about exposure levels, job histories, and duration of exposure. Some information is collected on the individual level, and each of these sources can be affected by measurement error. The resulting measurement error in the CE is therefore not simply described or estimated by random or Berkson error but a combination of the two, and the effect of this error is therefore not simply described. We therefore considered only the possible association between aspects of the exposure assessment strategy and the heterogeneity in K_L_ values and made no attempts to estimate the magnitude of the error in the CE directly.

The studies incorporated in the meta-analysis were different with regard to the definition of the reference population; an external reference population with only background exposure or an internal reference population with low exposure. By pooling potency estimates from the different studies, we made an implicit assumption that any potential exposure in the reference populations had no consequences for the reported estimates. Different steps were necessary to prepare the extracted data for this meta-analysis. A crucial step was assignment of specific exposure estimates to the unbounded upper CE categories. Category point estimates result in a loss of information because they are based on aggregated and not the original data on the individual level. Current approaches also ignored an expected log-normal distribution of exposure data within an exposure category. However, in most cases, data were not available to produce more optimal estimates. Another limitation of this meta-analysis is that we analyzed only five covariates, and other factors may be correlated with exposure–response slopes, such as sufficient latency. Similarly, other unknown factors may be correlated with quality of the exposure assessment that we did not assess, although we made efforts to explore the most prominent factors mentioned in the literature. Our ability to evaluate some exposure assessment covariates, particularly the more subjective ones (sufficient documentation and job histories), was limited by the availability of information provided by authors of the studies. It is noteworthy that publication requirements have evolved such that more transparent information was generally provided in more recent publications. The exclusion strategy based on quality criteria, although superior to weighting based on quality scores (Greenland and O’Rourke 2001), is not completely immune to bias. As expected, restriction to fewer studies resulted in generally wider CIs for the meta-K_L_ estimates (lower precision).

Several meta-analyses have been published on the risks of lung cancer and mesothelioma in asbestos-exposed workers. Hodgson and Darnton (2000) estimated “cohort average” R_L_ values based on a mean CE for each cohort, and derived exposure–response relationships across cohorts. This procedure allowed Hodgson and Darnton to use studies for which only a single estimate of average exposure was available, and they thus included more studies than any other of the earlier meta-analyses, irrespective of study quality. Hodgson and Darnton concluded that cohorts exposed only to crocidolite or amosite had quite similar exposure-specific risk levels, whereas chrysotile-exposed cohorts show a less consistent picture. They specifically pointed to the discrepancy between the mortality experience of the chrysotile-exposed cohorts of textile workers in South Carolina (Hein et al. 2007) and the miners and millers from Quebec (Liddell et al. 1997) and considered the South Carolina risk per unit of exposure to be unusually high. Our evaluation showed that the South Carolina textile worker study is among the studies with the highest-quality exposure assessment. We excluded the Quebec mine study in the analysis of only higher-quality studies because of a variety of limitations, notably insufficient job history information.

Berman and Crump (2008a, 2008b) analyzed exposure–response relationships within cohorts and estimated fiber-specific meta-risk estimates. They concluded that, for lung cancer, there is some evidence of larger K_L_ values from amphibole asbestos exposure, although there was considerable dispersion in the data. The Berman and Crump (2008b) analysis considered quality of the exposure assessment. They presented uncertainty intervals that reflected, in addition to statistical variation, other forms of uncertainty such as uncertainty in exposure estimates. However, they did not explicitly analyze how the quality of the exposure assessment affected the slope of the exposure–response relationship. They also pointed to the discrepancy in K_L_ values between the South Carolina textile plant and the Quebec mines. In their separate meta-analysis (Berman and Crump 2008a) they considered the fraction of the asbestos exposure in a given environment represented by chrysotile versus amphibole asbestos, long versus short fibers, and thin versus thick fibers, estimated from information in the literature for a particular environment in relation to the K_L_ values. For lung cancer, they found a significant difference in potency for chrysotile and amphibole asbestos for thin fibers (widths < 0.4 μm and < 0.2 μm) but not for thicker fibers (Berman and Crump 2008a), although their meta-analysis also suffered from limited statistical power (*n* = 15 studies).

The recent meta-analyses by Berman and Crump (2008a) and Hodgson and Darnton (2000) are more complete and exhaustive in their analysis and discussion of the characteristics of individual studies than many previous reviews, but they did not evaluate quality as extensively as we did for the present analysis. Lash et al. (1997) noted a significant correlation between K_L_ values and the maximum CE of a specific cohort. They concluded that this suggests that equivalent CEs reported for different cohorts represent different effective doses. Although this cannot be excluded, it seems more likely according to exposure misclassification theory that the signal-to-noise ratio for a specific cohort explains the association with K_L_ values. Studies with a high CE for the highest exposure category usually have higher ratios for the highest and lowest CE categories. Thus, high exposures may be indicative of a considerable signal for the cohort study. We found a difference in meta-K_L_ values between studies with high versus low exposure ratios in CE, although the difference was not statistically significant (0.20; 95% CI: 0.04, 0.35; and 0.10; 95% CI: –0.05, 0.26, respectively).

This meta-analysis has some important implications. Because study quality predicted heterogeneity in K_L_ values between studies to a large extent, (exposure assessment) study quality must be taken into account in risk assessments considering other potential determinants of heterogeneity such as fiber type and industry. As noted by Hodgson and Darnton (2000) and Berman and Crump (2008a), large differences exist between South Carolina textile and Quebec mine lung cancer potency factors (Hein et al. 2007; Liddell et al. 1997); a sensitivity analysis of the influence of a single study on meta-K_L_ values corroborated that these studies are most influential [Supplemental Material, Figure 2 (http://dx.doi.org/10.1289/ehp.1002879)]. It has been proposed that the discrepancy between these chrysotile-exposed cohorts may be explained by differences in fiber dimension distributions between these industries (Dement and Wallingford 1990; Gibbs and Hwang 1975). Animal studies have provided evidence that longer and thinner fibers may be more biologically active in generating respiratory disease (Davis and Jones 1988; Lippmann 1990; Stanton et al. 1981). A greater proportion of the asbestos aerosol is < 5 μm in length and < 0.25 μm in diameter, in the mining versus the textile industry (Dement et al. 2008), which could partially explain the low K_L_ reported for the Quebec mine cohort. TEM-based measurements have higher resolution than PCM-based measurements and allow for accurate characterization of fiber dimension distributions. Recent epidemiological studies that made use of TEM-based measurements generally found strongest associations with longer fibers (Loomis et al. 2010; Stayner et al. 2008). We acknowledge that fiber dimensions likely explain some variability in potency. However, exposure assessment limitations likely contributed to heterogeneity in study-specific potencies as well, because of attenuation of exposure–response relations (K_L_ values) resulting from varying degrees of misclassification of exposure. In our judgment, the South Carolina study among textile workers (Hein et al. 2007) was one of the studies with the fewest limitations in the exposure assessment methodology. This is in agreement with the observations by Berman and Crump (2008a). The Quebec mine cohort, in contrast, suffers from several exposure assessment limitations, such as incomplete job history information and undocumented moving between mines and mills; the exposure–response was relatively flat. The general population case–control study (Gustavsson et al. 2002) had the highest K_L_ value, and the South Carolina textile workers study was third (Hein et al. 2007). It is noteworthy that these two studies, which both involved predominantly exposure to chrysotile, are among the studies with the highest-quality exposure assessment and the highest K_L_ values.

The existing asbestos literature has numerous limitations. Many of the epidemiological studies are small and have low precision. Misclassification of exposure is more likely to contribute to imprecision and heterogeneity in observational studies than in experimental studies. Study-specific potency estimates are highly heterogeneous, and current and previous efforts (e.g., Berman and Crump 2008a, 2008b; Hodgson and Darnton 2000; Lash et al. 1997) to explain inconsistent potency estimates have only partially explained their variance. Furthermore, for several studies, exposure assessment was poorly integrated into the epidemiological design, for instance regulatory compliance versus representative sampling for epidemiological purposes, and absence of repeated sampling over time. Given the limitations of the data as it currently stands, we recommend that additional research be conducted on risks of asbestos to allow risk assessors to model attributable risk with greater confidence. Recent work on characterizing size distributions of old samples of Quebec mine and North and South Carolina textile plant fibers with TEM (Berman 2010; Dement et al. 2009, 2011) might illuminate reasons for discordant chrysotile potencies between cohorts. However, we recommend that prospective studies, or at least retrospective studies that involve comprehensive quantitative exposure assessment, be performed on cohorts, for instance, in India and other non-Western countries where large quantities of asbestos are still used.

## Conclusions

Asbestos–lung cancer risk relationships are highly heterogeneous, and factors describing the exposure assessment strategy seem to account for part of the disparity between studies’ lung cancer potency factors. Combining only higher-quality studies yields higher meta-estimates of lung cancer risk per unit of exposure than does including all available studies. Given these results, it is difficult to distinguish differences in potency between chrysotile and amphiboles for lung cancer, because too many studies have major limitations in the exposure assessment component. When analysis is restricted to studies with few quality limitations of the exposure assessment component, the epidemiological evidence base is too sparse to draw conclusions about potency differences per fiber type. Only further research will satisfactorily clarify the controversial issue of fiber-specific potencies and, furthermore, is warranted considering the politically sensitive nature of this question and the widespread public health impact of historic and current asbestos use. These results highlight that it is imperative to pay careful attention to the quality of the exposure assessment component of epidemiological studies on occupational and environmental risk factors. These results cast doubt on assertions that the epidemiological evidence for lung cancer strongly supports a difference in potency for different asbestos fiber types.

## Supplemental Material

(200 KB) PDF
