# Supplementary material for: A Meta-analysis of Asbestos and Lung Cancer: Is Better Quality Exposure Assessment Associated with Steeper Slopes of the Exposure–Response Relationships?
Source: Environ Health Perspect. 2011 Jun 27;119(11):1547–55. doi: 10.1289/ehp.1002879 (PMC3226488; doi:10.1289/ehp.1002879)
Supplement: (200 KB) PDF [file ehp.1002879.s001.pdf]

Supplemental Material

A Meta-Analysis of Asbestos and Lung Cancer: Is Better Quality Exposure Assessment Associated with Steeper Slopes of the Exposure-Response Relationships?

Virissa Lenters[1], Roel Vermeulen[1,2], Sies Dogger[3], Leslie Stayner[4], Lützen Portengen[1], Alex Burdorf[5], Dick Heederik[1,2]

[1] Utrecht University, Institute for Risk Assessment Sciences, Division of Environmental Epidemiology, Utrecht, The Netherlands

[2] Julius Center for Health Studies and Primary Care and Public Health, University Medical Center Utrecht, Utrecht, The Netherlands

[3] Health Council of the Netherlands, The Hague, The Netherlands

[4] University of Chicago, Department of Epidemiology and Biostatistics, Chicago, USA

[5] Erasmus University Rotterdam, Rotterdam, Department of Public Health, The Netherlands

Table of Contents

	Page
**Extracted Data**	
**Table 1.** Detailed descriptive information of the cohort and case-control studies included in the meta-analysis	3
**Appendix 1.** Abstracted and calculated exposure-response data, and a brief description of the (documented) job history information, for each study included in the meta-analysis	5
**Alternate Exclusion Analysis**	
**Table 2.** Results from the random effects meta-analysis in which studies were excluded stepwise, based on the number of exposure assessment quality criteria they failed to satisfy	14
**Sensitivity Analyses**	
**Table 3.** Comparison of lung cancer potency estimates from the current study, Berman and Crump (2008a,b) and Hodgson and Darnton (2000)	15
**Table 4.** Sensitivity analysis comparing results from the random effects meta-analysis in which studies were eliminated stepwise with specific exposure assessment descriptors, using potency estimates from the current study, Berman and Crump (2008a, 2008b) and Hodgson and Darnton (2000)	17
**Table 5.** Sensitivity analysis comparing results from the random effects meta-analysis in which studies were eliminated stepwise with specific exposure assessment descriptors, using K_L values calculated with the uppermost cumulative exposure category omitted, and the regression line forced through an intercept of 1	18
**Table 6.** Sensitivity analysis comparing random effects meta-analysis and subgroup analyses using potency estimates from the current study, Berman and Crump (2008b) and Hodgson and Darnton (2000)	19
**Table 7.** Sensitivity analysis comparing random effects meta-analysis and subgroup analyses using K_L values calculated with the uppermost cumulative exposure category omitted, and the regression line forced through an intercept of 1	20
**Figure 1.** Funnel plot to assess potential publication bias	21
**Figure 2.** Exclusion sensitiviy plot	22
**References**	23

Supplemental Material, Table 1. Detailed descriptive information of the cohort and case-control studies included in the meta-analysis

	Cohort	Primary reference	N	Fibre type	CF	Cumulative exposure categories (f-yr/ml)		Recruitment	Follow-up period	Exposure duration	Impinger/other measurements (year)	PCM (year)	Measurement coverage (%)		Lagged CE
						Mean of lowest; highest	Ratio highest: lowest						Total	PCM	
1	Quebec mines and mills	Liddell et al. 1997	~11000	Chry	I	4.71; 4710	1000	born 1891-1920	1904-92	~1904-76	1948-66	≥1969	~25%	10%	CE to age 55
2	Italian mine and mill (Balangero)	Pira et al. 2009	1056	Chry	N/A	50; 666.7	13	1946-87	1946-2003	1916-90	Simulation of earlier conditions	≥1969	24%	24%	CE
3	Connecticut friction products plant	McDonald et al. 1984	3513	Chry	E	15; 400	27	1913-59	1939-77	1913-77	1930, 35, 36, 39	>1969	~30%	11%	CE
4	South Carolina textile plant	Hein et al. 2007	3072	Chry	I	0.75 ; 200	267	1940-65	1940-2001	1896-77	1930-71	≥1965	>58%	15%	CE10
5	North Carolina textile plants	Loomis et al. 2009	5770	Chry	I	0.383; 408.3	1066	1950-73	2003	<1925->1994	1935-71	1964-86	>74%	>32%	CE10
6	Wittenoom, Australia, mines and mills	Berry et al. 2004	6358	Croc	N/A	0.11; 219.9	1999	1943-66	1943-2000	1937-66	1948-58 (ignored)	1966 survey	< 5%	< 5%	CE
7	Paterson, NJ, insulation manufacture	Seidman et al. 1986	820	Am	N/A	3; 416.7	139	1941-45	1941-82	1941-54	-	No factory meas.[b]	0%	0%	CE
8	Tyler, Texas, insulation manufacture	Levin et al. 1998	1121	Am	N/A	11.25; 375.00	33	1954-72	1954-93	1954-72	-	1967, 70, 71	~25%	~25%	CE
9	Libby, Montana, Vermiculite mines and mills	Sullivan 2007	1672	Tre	I	2.25; 167	74	1935-81	2001	1935-90	1956-69	1967-82	47%	27%	CE10
10	British friction products factory (Ferodo)	Berry and Newhouse 1983	13460	Mix	N/A	4.5; 228	51	1941-77	1942-79	1910-79	Simulation of earlier conditions	≥1967	19%	19%	CE
11	Ontario cement factory	Finkelstein 1984	740	Mix	E	15; 250	17	1948-59	1977 or 1981	1948-77	1949-79	≥1969	>80%	28%	CE
12	New Orleans cement plants [a]	Hughes et al. 1987	6931	Mix	I	4.2; 256.2	61	Plant 1: 1942-69; Plant 2: 1937-69	1937-82 or age 80	1937-72	1952-69	≥1969	61%	9%	CE10
13	Swedish cement plant	Albin et al. 1990	2898	Mix	I	3.1; 88.2	28	1907-77	1927-86	1907-77	1956-69	≥1969	30%	11%	CE

Supplemental Material, Table 1 – *continued.*

	Cohort	Primary reference	N	Fibre type	CF	Cumulative exposure categories (f-yr/ml)		Recruitment	Follow-up period	Exposure duration	Impinger/other measurements (year)	PCM (year)	Measurement coverage (%)		Lagged CE
						Mean of lowest; highest	Ratio highest: lowest						Total	PCM	
14	Belgium cement plant	Laquet et al. 1980	1973	Mix	N/A	25; 2000	80	1963-77	1963-77	1928-77	No meas., estimated back to 1928	1970-76	12%	12%	CE
15	U.S. factory retirees (Johns Manville)	Enterline et al. 1987	1074	Mix	E	186; 2928	16	retired 1941-67	1941-80	1890-1980	mid-1950s	-	~30%	0%	CE
16	U.S. & Canada insulation workers	Selikoff and Seidman 1991	17800	Mix	E	37.5; 375	10	Joined union 1967	1967-86	~1920-86	-	-	0%	0%	CE10
17	Pennsylvania textile plant	McDonald et al. 1983	4024	Mix	E	15; 330	22	1959	~1920-77	~1900-67	1930-39, ≥1956	≥1967[c]	~55%	0%	CE10
18	Rochdale, England textile plant	Peto et al. 1985	3211	Mix	I	5.92; 256.57	43	1933-74	1953-83	1933-78	1951-64	≥1965	60%	29%	CE5
19	Stockholm County population	Gustavsson et al. 2002	1038 cases, 2359 referents	Mix	N/A	0.0; 8.80	>100	1950-1990 lived in city	1985-90 cases identified	~1925-74	-	1969-73	10%	10%	CE

Predominant fiber type: Chry, Chrysotile; Croc, Crocidolite; Am, Amosite; Tre, Tremolite; Mix, Mixed.
CF, Conversion factor; indicates whether measurements of particles (mppcf) were converted to fibers/ml with an I (internally) or E (externally) derived conversion factor based on paired measurements or a generic factor, respectively. N/A (not-applicable) denotes that no conversion factor was applied because exposures estimates were based on PCM-based estimates, and were expressed in units of f-ml/yr.
Measurement coverage indicates what proportion of the exposure time was covered by a) total, or any sampling, and b) PCM-based exposure assessment
Lagged CE indicates whether exposures in the CE(x) years previous to follow-up were discarded.
[a] Results for Hughes et al., 1987, originally stratified by fiber type, were combined for this meta-analysis
[b] Estimated based on measurements taken between 1967-71 at similar plants in Texas and Pennsylvania (of the same company making the same products with the same machinery, fiber type and production processes (Seidman et al. 1986))
[c] Survey data seems to have not been used

Supplemental Material, Appendix 1. Abstracted and calculated exposure-response data, and a brief description of the (documented) job history information, for each study included in the current meta-analysis

Abbreviations
CE, cumulative exposure (to asbestos); SMR, standardized mortality ratio; RR, relative risk or rate ratio; OR, odds ratio; Obs., observed lung cancer cases/deaths; Exp., expected lung cancer cases/deaths; PY, person years; LCL, lower limit and UCL, upper limit of 95% confidence interval

The following data were used to derive the alpha (α) and K_L values, and associated standard errors, for each study using the model:

$$RE = \alpha\,(1 + K_L * CE)$$

where RE = risk estimate (SMR, RR, or OR)
α = intercept
K_L = lung cancer potency factor of asbestos
CE = cumulative exposure to asbestos (lagged 10 years if data provided, otherwise unlagged CE). Midpoints of the CE categories were used unless otherwise specified below.

1. Quebec mines and mills

CE (mpcf.y)[a]	CE midpoint (f-yr)/ml[b]	SMR[a]	Obs.[a]	Exp.[c]
<3	4.71	1.12	75	66.96
3, <10	20.41	1.27	64	50.39
10, <30	62.8	1.03	61	59.22
30, <60	141.3	1.32	60	45.45
60, <100	251.2	1.45	61	42.07
100, <200	471	1.27	67	52.76
200, <300	785	1.10	35	31.82
300, <400	1099	1.46	29	19.86
400,<1000	2198	1.84	88	47.83
≥1000	4710	2.97	47	15.82

[a] Data from Liddell et al. (1997), Table 8
[b] Data from Berman and Crump (2008b), Appendix B, Table B1
[c] Calculated as Exp. = Obs./SMR

Job histories: Insufficient
There are some major deficiencies in the job history data for this cohort. The Quebec cohort includes workers from 1 large mine and mill company in Asbestos, Quebec, and 7 other large to small operations in or near Thetford Mines. Personnel records were transcribed onto cards starting in 1966, including data on dates of employment and the payroll record of each job and mine worked in, and periods of leave. However, “Work histories were incomplete for at least 560 men who had worked at one company whose records were not transferred when the ownership of the company changed in 1964, also for a small number of others who had been employed both by this company and others” (Liddell et al. 1997). In addition, Liddel et al. (1997) acknowledge that “Many employees in the more recently established companies had worked previously elsewhere in the industry and often this was not indicated in the extant records. There were also frequent unrecorded movements of personnel between the mine and mill and the factory at Asbestos.”

2. Italian mine and mill (Balangero)

CE (fibre-years) [a]	CE midpoint	SMR[a]	Obs.[a]	Exp.[b]
<100	50	0.83	9	10.84
100-400	250	1.57	17	10.83
≥400	666.7	1.37	19	13.87

[a] Data from Pira et al. (2009), Table 2
[b] Calculated as Exp. = Obs./SMR

Job histories: Sufficient
Dates of employment and job categories were obtained from the factory personnel records (Piolatto et al. 1990; Pira et al. 2009; Rubino et al. 1979). "Individual details of jobs while in employment were obtained from factory records and checked where possible by asking colleagues still at work. … Almost all workers had changed their job during their working life at the factory. An attempt has therefore been made to quantify individual exposure by calculating, for each worker, an approximate value for the accumulated dose of inhaled fibres." (Rubino et al. 1979)

3. Connecticut friction products plant

Range[a] (mppcf-yr)	CE (f-ml/y)[b]	SMR[a] /100	Obs.[a]	Exp.[c]
<10	15	1.674	55	32.86
10-20	45	1.017	6	5.90
20-40	90	1.054	5	4.74
40-80	180	1.628	6	3.69
≥80	400	0.5522	1	1.81

[a] Data from McDonald et al. (1984), Table 5
[b] Berman and Crump (2008b), Appendix B applied a conversion factor of 3 f/ml per mpcf; upper midpoint calculated as 5/3*lower bound for the uppermost CE category
[c] Calculated as Exp. = Obs./SMR

Job histories: Insufficient
Data on job histories abstracted from personnel records were only available at the department level, and generally not by job or process. "In some departments there was one very dusty process on which few employees worked and other less dusty processes on which many employees worked. The more dusty process had to be taken into account in estimating departmental dust levels, which may have resulted in some general overestimation of exposures for most employees in these departments and underestimation for a few." (McDonald et al. 1984)

4. South Carolina textile plant

Range[a]	CE	SMR[a]	Obs.[a]	Exp.[a]	LCL[a]	UCL[a]
<1.5	0.75	1.54	34	22.1	1.07	2.15
1.5-<5	3.25	1.30	33	25.3	0.90	1.83
5-<15	10	1.57	34	21.7	1.08	2.19
15-<60	37.5	1.86	35	18.8	1.30	2.59
60-<120	90	4.02	37	9.2	2.83	5.53
≥120	200[b]	5.36	25	4.7	3.47	7.92

[a] Data from Hein et al. (2007), Table 3 (CE lag 10 years)
[b] Upper midpoint calculated as 5/3*lower bound for the uppermost CE category

Job histories: Sufficient
Detailed job histories of employment dates, department(s) and job(s) held by the worker were available from 1930 onwards. Records also contained dates when workers were absent, terminated and rehired. Individual exposure was estimated for each day of employment based on an operation- and calendar year-specific job-exposure matrix. (Dement et al. 1983a, 1983b; Hein et al. 2007)

5. North Carolina textile plants

Range [a]	CE [b]	RR [a]	Obs. [a]	Exp.	LCL [a]	UCL [a]
<2.3	0.38	1 [d]	37	37.00		
2.3-<11.5	5.69	1.13	37	32.74	0.71	1.78
11.5-<34.8	20.97	1.58	35	22.15	0.99	2.53
34.8-<152.7	75.56	1.25	37	29.60	0.79	2.00
>152.7	408.34	1.88	35	18.62	1.14	3.08

RR, rate ratio (internal poisson regression)
[a] Extracted from Table 5 of Loomis et al. (2009), CE lagged 10 years
[b] Midpoints obtained via direct communication with the authors
[d] Lowest category (reference category) ignored in calculating the alpha and K_L values

Job histories: Insufficient
The authors acknowledge that exposure assessment was hampered by limitations in job history data. A plant-, department-, job- and time-specific job exposure matrix was developed (Dement et al. 2009). Scanned personnel records were obtained from the United States Public Health Service (USPHS) for workers hired before 1968, and by a review of records at 3 of the 4 plants still open for workers hired after 1968. Missing information on job titles for some workers employed in the 1970s and 1980s was available from the medical records of the Dusty Trades Surveillance Program. Few work histories available prior to 1935. “Approximately 27% of the work history records available for exposure-response analysis were missing details of jobs held within departments” (Loomis et al. 2009). For these records, exposure estimates were modelled. Workers for whom only a plant average could be estimated were excluded; “contrasts in estimated exposure between workers may have been reduced to an unknown degree by retaining workers with complete information about departments, but not job titles” (Loomis et al. 2009).

6. Wittenoom, Australia mines and mills

Range [a]	CE Midpoint	CE [a]	SMR [c]	Obs. [a]	Exp. [a]
0-1	0.5	0.11	2.62	50	19.1
1-5	3.0	2.65	2.65	65	24.5
5-10	7.5	7.03	3.61	53	14.7
10-30	20.0	17.7	3.03	57	18.8
30-60	45.0	42.8	2.63	25	9.5
60-120	90	84.3	4.75	29	6.1
>120	200 [b]	219.9	4.89	23	4.7

[a] Data from Berman and Crump (2008b), Table B9, who obtained raw data from de Klerk (Berry et al. 2004)
The CE values are not midpoints of the categories. However, since Berman and Crump (2008b) state that they have raw data from de Klerk, we used these “average” values instead of calculating the midpoints.
[b] Upper midpoint calculated as 5/3*lower bound for the uppermost CE category
[c] Calculated as SMR = Obs./Exp.

Job histories: Sufficient
Employment records from the Astralian Blue Asbestos Company were obtained, and supplemented by information from the Perth Chest Clinic and Western Australian Mineworkers Relief Fund. Individual data on job histories (for 87 possible job categories) was available, although it is not clear how complete this data was. (Armstrong et al. 1988; Berry et al. 2004; de Klerk et al. 1989)

7. Paterson, New Jersey insulation manufacture

Range[a]	CE	SMR[b]	Obs.[a]	Exp.[a]
<6	3	2.83	15	5.3
6-11.9	9	4.14	12	2.9
12-24.9	18.5	4.41	15	3.4
25-49.9	37.5	4.64	13	2.8
50-99.9	75	7.08	17	2.4
100-149.9	125	6.00	9	1.5
150-249.9	200	11.54	15	1.3
>250	416.7[c]	16.67	15	0.9

[a] Data from Seidman et al. (1986)
[b] Calculated as SMR = Obs./Exp.
[c] Upper midpoint calculated as 5/3*lower bound for the uppermost CE category

Job histories: Sufficient
Dates of employment (duration, time period) were used for the first follow-up (Seidman et al. 1979). Information on jobs of the workers was added for the second follow-up to derive quantitative estimates of cumulative exposure to asbestos (see Table XIII, Seidman et al. 1986). The authors were not forthcoming as to how detailed or complete the job information was.

8. Tyler, Texas insulation manufacture

Range [a] (duration of exposure, years)	CE[b]	SMR [c]	Obs. [a]	Exp.[a]
<0.5	11.25	2.58	23	8.9
0.5-1	33.75	2.73	3	1.1
1-5	135	2.22	4	1.8
>5	375.0[d]	4.00	6	1.5

[a] Data from Levin et al. (1998), Table 2
[b] CE was estimated as in Berman and Crump (2008b); duration of exposure was multiplied by an assumed plant average fibre level of 45 fibers, as the range in fibre concentrations reported by Levin et al. (1998) for three surveys conducted in 1967, 1970 and 1971 was 15.9 to 91.4 fibres/ml.
[c] Calculated as SMR = Obs./Exp.
[d] Upper midpoint calculated as 5/3*lower bound for the uppermost CE category

Job histories: Sufficient
Employment records were available for workers, including temporary workers. Workers with missing employment dates were excluded (n=35 of 816). Cumulative exposure was estimated by multiplying duration of exposure by a plant average asbestos fiber level; authors do not mention records on jobs or processes performed by individual workers. (Levin et al. (1998)

9. Libby, Montana vermiculite mines and mills

Range[a] (fibers/cc-yr)	CE	SMR[b]	Obs.[a]	Exp.[a]	Obs.[d]	Exp.[d]
0-4.5	2.25	1.39	19	13.02	20	14.35
4.5-23	12	1.66	24	14.62	24	14.46
23-100	55	1.71	23	12.95	23	13.44
≥100	167[c]	2.06	23	11.93	26	12.64

[a] Data from Sullivan et al. (2007), Table 3 (CE lagged 15 years)
[b] Calculated as SMR = Obs./Exp.
[c] Upper midpoint calculated as 5/3*lower bound for the uppermost CE category
[d] Data from Berman and Crump (2008b), Appendix B, Table B13: values for CE lagged 10 years, based on personal communication with P. Sullivan by Berman and Crump.

Job histories: Sufficient
Work histories were abstracted from personnel and pay records, and were reabstracted for the latest follow-up. Information on job assignments was available, and a job-exposure matrix developed specifically for this cohort was used. (Amandus et al. 1987; Sullivan et al. 2007)

10. British friction products factory (Ferodo)

Range[a]	CE	OR[a]	Exp.[b]	Cases[a]	Controls[a]
0-9	4.5	1.00[c]	50.00	50	132
10-49	29.5	0.79	46.84	37	124
50-99	74.5	0.86	15.12	13	40
100-356	228	0.88	5.68	5	15

[a] Data from Berry and Newhouse (1983), Table 14
[b] Expected deaths = cases/odds ratio
[c] Lowest category (reference category) ignored in calculating the alpha and K_L values

Job histories: Sufficient
Dates of employment were obtained from personnel records. "The actual job was not recorded explicitly but the "cost-centre" of the work was coded… job histories were extracted only for groups of special interest." The cohort was restricted to those workers who had started after the personnel records were instituted (in 1941). Cumulative exposures were derived using work histories. (Berry and Newhouse 1983)

11. Ontario cement factory

Range[a]	CE	SMR[c]	Obs.[a]	Exp.[d]	Mortality rate[a]
≤30	15	2.31	3	1.3	3.0
30.1-75	52.5	6.00	6	1.0	8.0
75.1-105	90	12.50	5	0.4	15.7
105.1-150	127.5	8.33	5	0.6	11.7
>150	250[b]	2.86	2	0.7	3.5

[a] Data from Finkelstein (1984), Table 7
[b] Upper midpoint calculated as 5/3*lower bound for the uppermost CE category
[c] Calculated as SMR = Obs./Exp.
[d] Data from Berman and Crump (2008b), Appendix B, Table B14 (Exp.=Obs./mortality rate)

Job histories: Insufficient
Employment records, including job assignments were available. However, cumulative exposures were only calculated for the production workers (n=428). "It was, unfortunately, not possible to calcualte exposures for the maintenance workers because of inadequate data" (n=107). There were also internal controls from the factory who were from the rock wool/fiber glass or other minimal exposure areas, or who had been exposed only after 1961 (n=205). (Finkelstein 1984)

12. New Orleans cement plants

Plant	Range (mppcf-yr)[a]	Mean[a]	CE[c]	SMR[d]	Obs.[a]	Exp.[a]
1	<6	4	5.6	1.03	3	2.9
	6-24	13	18.2	1.13	9	8.0
	25-49	35	49.0	0.54	2	3.7
	50-99	74	103.6	0.79	3	3.8
	≥100	183	256.2	1.22	5	4.1
	Range[b]	Mean[b]	CE[c]	SMR[d]	Obs.[b]	Exp.[b]
2	<3	3	4.2	1.06	20	18.9
	3-5	12	16.8	1.36	19	14.5
	6-24	36	50.4	2.00	12	6.0
	25-49	71	99.4	1.82	10	5.5
	≥50	164	229.6	2.31	12	5.2

[a] Data from Hughes et al. (1987), Table 8
[b] Data from Hughes et al. (1987), Table 9
[c] A conversion factor of 1.4 (Hammad et al. 1979) was used to convert mppcf-yr values to units PCM f/cc-yr.
[d] Calculated as SMR = Obs./Exp.
NOTE: Results were originally stratified by plant (which differ in predominant fiber type used) and were combined (stacked) for the purpose of calculating one K_L value for this cohort

Job histories: Insufficient
This cohort is comprised of workers from two plants. The proportion of workers exposed to amphiboles versus chrysotile and exposure levels differed between the plants. Personnel records provided information on dates of employment. Social Security Administration records were checked to verify how many employees were identified from company records for Plant 2 (not possible for plant 1); 96% were verified. Detailed work history data on specific jobs or tasks performed was not available; “there was only limited variability in recorded job titles in this plant [1]” (55% were listed only as "labourer"). Workers at plant 2 were crudely categorized into two groups: those who worked in areas likely to have involved exposure to amphiboles (pipe production), and those not likely to have worked with amphiboles. With respect to analysing dose-response relations, the authors admit “The accuracy of job records in reflecting actual work area and exposure to fibre is critical to this analysis but cannot be assessed.” (Hughes et al. 1987)

13. Swedish cement plant

Range[a]	Median[a]	CE Mean[a]	Obs.[b]	RR[a]	LCL[a]	UCL[a]
0-15	1.4	3.1	19	1.8	0.8	3.9
15-39	24.2	25.6	5	1.9	0.7	5.3
≥40	67.0	88.2	3	1.9	0.5	7.1

RR, relative risk
[a] Data from Albin et al. (1990), Table 4
[b] Calculated based on total deaths presented in Albin et al. (1990): 27 from malignant respiratory disease, non-mesothelioma

Job histories: Insufficient
Job histories were obtained from personnel records, although for 22% job history data was not available, and only the first assignment was known for some other workers (Albin et al. 1990).

14. Belgium cement plant

Range[a]	CE	SMR[c]	Obs.[a]	Exp.[a]	Internal controls[b]	Cases/ Total[b]
0-49	25	1.16	6	5.16	20	0.23
50-99	75	1.24	3	2.43	17	0.15
100-199	150	1.09	5	4.60	7	0.42
200-399	300	0.54	4	7.47	32	0.11
400-799	600	0.52	1	1.95	8	0.11
800-1599	1200		2	0.57	4	0.33
1600-3200	2400		0 [d]	0.17	0	-
800-3200 [e]	2000	2.70	2	0.74	4	

[a] Data from Lacquet et al. (1980), Table 8 (Expected deaths based on yearly mortality rates for Belgium.)
[b] Data from Lacquet et al. (1980), Table 9 (matched internal controls)
[c] Calculated as SMR = Obs./Exp.
[d] The one pleural mesothelioma case was ignored (only lung cancer cases considered in this study)
[e] The upper two CE categories were combined due to zero lung cancer cases in the uppermost category

Job histories: Sufficient
Information was available on in which area (of five areas) employees worked and when. Authors do not explicitly state how complete the work history data was (Lacquet et al. 1980).

15. U.S. factory retirees (Johns Manville)

Range[a]	Mean CE[a]	CE	SMR[b]	SMR[a]	Obs.[a]	Exp.
<125	62	186	1.82	182.3	23	12.6
125-249	182	546	2.03	203.1	14	6.9
250-499	352	1056	3.20	322.0	24	7.5
500-749	606	1818	4.00	405.0	10	2.5
≥750	976	2928	7.27	698.7	8	1.1

[a] Data from Enterline et al. (1987), Table 4
[b] Calculated as SMR = Obs./Exp.

Job histories: Sufficient
Data on jobs performed and time periods of jobs was available from personnel records (Enterline et al. 1987; Henderson and Enterline 1979). Jobs were placed in one of six classes or ranges. “At the time data for this study was collected the industrial hygienist assigned each worker to a principal department and a primary type of asbestos to which he had been exposed” (Enterline et al. 1987).

16. U.S. (& Canada) insulation workers

Years from onset[a]	CE[b]	SMR[c]	Obs.[a]	Exp.[a]
<15	37.5	1.79	7	3.87
15-19	112.5	2.93	34	11.62
20-24	187.5	3.09	85	27.47
25-29	262.5	3.69	172	46.62
30-34	337.5	4.38	252	57.36
35-39	375	4.13	193	46.75
40-44	375	4.18	129	30.79
45-49	375	3.51	66	18.81
50+	375	2.79	71	25.38
35+[c]	375[d]	3.77[c]	459[c]	121.8[c]

[a] Data from Selikoff and Seidman (1991), Table 4
[b] Data from Berman and Crump (2008b), Table B19; assuming an average duration of exposure of 25 years and exposure levels of 15 f/ml, as was performed in the EPA 1986 update based on data in Nicholson 1976,
[c] Calculated as SMR = Obs./Exp.
[d] Collated the CE 375 Obs. and Exp. categories, and calculated a K_L based on one 375 upper category

Job histories: Sufficient
Data on "date of onset of insulation work, and employment history" was abstracted from the insulators' union records (Selikoff and Seidman 1991). Rather than the more standard duration of exposure, the authors reported 'years from onset of employment', which is not optimal for deriving quantitative estimates of cumulative exposure. (Nicholson 1976; Selikoff et al. 1979)

17. Pennsylvania textile plant

Range[a]	CE	SMR[a]	SMR[b]	Obs.[a]	Exp.
<10	15	66.9	0.67	21	31.4
10-20	45	83.6	0.84	5	6.0
20-40	90	156.0	1.56	10	6.4
40-80	180	160.0	1.58	6	3.8
≥80	330	416.1	4.23	11	2.6

[a] Data from McDonald et al. (1983), Table 5
[b] Calculated as SMR = Obs./Exp.

Job histories: Sufficient
Data on dates of employment and department were available from employment histories (McDonald et al. 1983). It is not clear whether information on department-specific jobs was available or used.

18. Rochdale, England textile plant

Cumulative dose (p ml^{-1} yr)[a]	Mean dose (p ml^{-1} yr)[a]	CE[b]	SMR[c]	Obs.[a]	Exp.[a]
<1000	209	5.92	1.15	34	29.53
1000-	1409	39.92	1.04	8	7.66
2000-	2511	71.13	1.67	11	6.60
3000-	3474	98.41	1.05	6	5.66
4000-	4551	128.92	2.33	10	4.29
≥5000	9057	256.57	2.22	24	10.83

[a] Data from Peto et al. (1985), Table 16
[b] The authors report a conversion factor of 35.3 particles per fiber
[c] Calculated as SMR = Obs./Exp.

Job histories: Sufficient
Detailed employment data was available for each worker, including in which section he had worked, the type of work, the detailed occupation (the actual machine used, if specified, or category of occupation), and whether the job was scheduled (Peto et al. 1985).

19. Stockholm County population-based

Exposure (fiber-years)[a]	Mean CE[a]	RR[b]	Cases[a]	Referents[a]	LCL[c]	UCL[c]
0	0		830	2020		
>0-0.99	0.56	1.195	95	188	1.082	1.321
1-2.49	1.51	1.447	70	104	1.176	1.780
2.5-4.49	3.44	1.819	25	28	1.301	2.544
≥4.5	8.80	2.500	18	19	1.496	4.178

[a] Data from Gustavsson et al. (2002), Table 2
[b] Calculated based on formula provided by authors: The relative risk (RR) at a cumulative dose of *x* fiber-years = $1.494^{\ln(x+1)}$; [c] 95%CI, substitute 1.494 with 1.193 and 1.871

Job histories: Sufficient
Information on lifetime occupational history was obtained from the study subjects, or from next of kin, via questionnaire. “The occupational history included company name and location, occupation, and work tasks for each work period of at least 1 year during the subject’s lifetime." (Gustavsson et al. 2002).

Supplemental Material, Table 2. Results from the random effects meta-analysis in which studies were excluded stepwise, based on the number of exposure assessment quality criteria[a] they failed to satisfy

Exclusion	No. of studies	I^2	Meta-α	95% CI	Meta-K_L*100	95% CI	AIC	Studies included
All 19 studies (0-5 criteria failed)	19	64.1%	1.48	1.14–1.81	0.13	0.04–0.22	28.2	1-19
≥ 3 criteria failed	10	63.8%	1.73	1.26–2.21	0.11	-0.02–0.24	30.5	1, 2, 3, 6, 7, 11, 13, 15, 16, 17
≤ 2 criteria failed	9	62.5%	1.24	0.79–1.69	0.19	0.03–0.34	30.5	4, 5, 8, 9, 10, 12, 14, 18, 19
≤ 1 criteria failed	4	77.4%	1.29	0.58–2.01	0.28	0.06–0.51	28.3	4, 5, 9, 18
0 criteria failed	2	88.4 %	1.42	0.40–2.44	0.55	0.11–0.99	25.3	4, 9

[a] The five criteria included 1) sufficient documentation, 2) ratio of highest : lowest CE midpoint > 50, 3) conversion factor internal, (versus generic, or based on external data), 4) coverage of exposure data >30% of exposure history, and 5) sufficient job histories.

Supplemental Material, Table 3. Comparison of lung cancer potency estimates used in the current study, Berman and Crump (2008a, 2008b) and Hodgson and Darnton (2000)

			Current study			B&C (2008b)[a]					B&C (2008a)[b]			H&D (2000)[c]				
No.	Cohort	Primary Reference (for the current study)	Alpha (α)	K_L *100	SE	B&C study no.[d]	Alpha[e]	K_L*100	90% CI		K_L*100	LB	UB	H&D study no.	Fiber[c]	R_L[f]	95% CI	
1	Quebec mines and mills	Liddell et al. 1997	1.15	0.03	0.01	CM1	1.15	0.029	0.019	0.051	0.029	0.0085	0.11	6	y	0.06	0.042	0.079
2	Italian mine and mill (Balangero)	Pira et al. 2009	1.02	0.07	0.09	CM3	0.937	0.051	0	0.57	0.051	0	1.1	10	y	0.03	-0.11	0.24
3	Connecticut plant	McDonald et al. 1984	1.62	-0.15	0.09	CF4	1.49	0	0	0.61	0	0	2.2	16	y	0.80	0.029	1.8
4	South Carolina plant	Hein et al. 2007	1.34	1.64	0.43	CT6	1.35	1.8	1.1	3.7	1.8	0.75	5.6	2f	y	6.7	3.6	11
4.1	South Carolina plant	-	-	-	-	CT6	1.07	1[h]	0.44	2.5	-	-	-	2m	y	4.6	2.9	6.7
5	North Carolina plants	Loomis et al. 2009	1.24	0.12	0.08	-	-	-	-	-	-	-	-	-	-	-	-	-
6	Wittenoom, Austr. mine	Berry et al. 2004	2.82	0.40	0.18	RM18	2.81 (2)	1.1	0.75	5.3	1.1	0.17	23	1	o	3.4	1.9	5.2
7	Paterson, New Jersey	Seidman et al. 1986	3.33	1.06	0.37	AI19	3.32 (2)	2.4	1.8	7.6	2.4	0.52	27	12	a	5.8	4.4	7.4
8	Tyler, Texas factory	Levin et al. 1998	2.49	0.13	0.18	AI20	2.48 (2)	0.28	0	2.2	0.28	0	6.6	-	-	-	-	-
9	Libby, Montana	Sullivan 2007	1.50	0.23	0.22	TM21	1.50	0.26	0	1.3	-	-	-	-	-	-	-	-
9.1	Libby, Montana	-	-	-	-	TM21	-	0.36[i]	0.03	3.6	-	-	-	-	-	-	-	-
10	British factory (Ferodo)	Berry and Newhouse 1983	0.78	0.07	0.28	MF7	Not pos.	0.058	0	0.8	0.058	0	1.8	17	yo	0	-0.36	0.36
11	Ontario factory	Finkelstein 1984	4.89	0.08	0.42	MP8	4.26 (2)	1.9	1.2	6.8	1.9	0.20	43	4	yo	5.2	2.7	8.8
12	New Orleans plants[g]	Hughes et al. 1987	1.14	0.25	0.20	MP9	1.14[g]	0.25	0	0.70	0.25	0	1.6	5a	ya	0	-0.53	0.54
12.1	New Orleans plants[g]	Hughes et al. 1987	-	-	-	-	-	-	-	-	-	-	-	5o	yo	0.81	0.21	1.6
12.2	New Orleans plants[g]	Hughes et al. 1987	-	-	-	CP5	1.14[g]	0.25	0	0.70	0.25	0	1.6	5y	y	1.3	-0.29	3.4
13	Swedish plant	Albin et al. 1990	1.81	0.08	0.77	MP10	1.82	0.067	0	6.4	0.067	0	26	15	yao	6.2	-0.77	21
14	Belgium factory	Laquet et al. 1980	0.87	0.03	0.07	MF11	0.924	0.0068	0	0.21	-	-	-	-	-	-	-	-
15	U.S. retirees (Johns Manville)	Enterline et al. 1987	1.42	0.11	0.06	MF13	1.43	0.11	0.041	0.28	-	-	-	3	yao	0.21	0.14	0.30
16	U.S./Canada insulation workers	Selikoff and Seidman 1991	2.39	0.18	0.09	MI15	2.39 (2)	0.28	0.25	0.93	0.28	0.045	5.1	8	yao	0.53	0.48	0.58
17	Pennsylvania plant	McDonald et al. 1983	0.52	1.83	0.95	MT16	0.519	1.8	0.27	4.5	1.8	0.07	16	11	ya	0.8	0.16	1.6
18	Rochdale, UK plant	Peto et al. 1985	1.10	0.42	0.22	MT17	1.10	0.41	0.12	0.87	0.41	0.046	2.3	9	yo	0.37	0.10	0.70
19	Stockholm, Sweden	Gustavsson et al. 2002	1.13	15.50	7.33	-	-	-	-	-	-	-	-	-	-	-	-	-
20	Vocklabruck, Austria	Neuberger et al. 1990	-	-	-	-	-	-	-	-	-	-	-	7	yo	0.45	-0.72	1.9
21	South Africa mines	Sluis-Cremer et al.1992	-	-	-	-	-	-	-	-	-	-	-	13a	a	1.9	-0.44	5.1
22	South Africa mines	Sluis-Cremer et al.1992	-	-	-	-	-	-	-	-	-	-	-	13o	o	5.2	0.71	12
23	Massachusetts	Talcott et al. 1989	-	-	-	-	-	-	-	-	-	-	-	14	o	10	3.9	21

B&C, Berman and Crump; H&D, Hodsgon and Darnton

Supplemental Material, Table 3 – *continued.*

Primary references listed for current study; older publications were used by B&C 2008 and H&D 2000 for some cohorts. Additional studies included by B&C 2008b and/or H&D 2000 but not included in the current study are numbered 20 to 26. Exposure assessment characteristics were not evaluated and classified for the studies (no. 20-26) not included in the current paper (Lenters et al. 2011).

[a] Data extracted from Table 3. Berman DW, Crump KS. 2008b. Update of potency factors for asbestos-related lung cancer and mesothelioma. Crit Rev Toxicol 38(S1):1-47. (2) indicates that the alpha was truncated to a maximum value of 2.

[b] Data extracted from Table 2. Berman DW, Crump KS. 2008a. A meta-analysis of asbestos-related cancer risk that addresses fiber size and mineral type. Crit Rev Toxicol 38(S1):49-73. (n=15 unique cohorts) The K_L extracted was the "Best" K_L (as labelled in Table 2).
Note: LB and UB are the 'Uncertainty Interval' formed by combining the 90% confidence interval with uncertainty factors described in Berman and Crump (2008a) Table 1 (or A-3 (B&C 2008b))

[c] Data extracted from Hodgson JT, Darnton A. 2000. The quantitative risks of mesothelioma and lung cancer in relation to asbestos exposure. Am Occup Hyg 44:565-601. y, a and o represent chrysotile, amosite and crocidolite exposures respectively

[d] First digit of code: A = amosite; C = chrysotile; M = mixed fibers; R = crocidolite; T = winchite-richerite (2nd digit = industry)

[e] Alphas from Berman and Crump 2008b (Appendix); (2) indicates that these alphas were truncated to 2

[f] R_L is the percentage excess of expected lung cancer mortality per unit of cumulative exposure; this 'cohort average' risk is calculated based on the formula $R_L = 100(O_L-E_L)/(E_L X)$ where O_L and E_L are the numbers of observed and expected lung cancers, respectively and X is the cohort mean exposure.

[g] K_Ls for the cohorts from New Orleans plants were collated for the current study as some workers in both plants likely were exposed to amphiboles (specifically, crocidolite for some workers at Plant 2). Berman and Crump (2008) reported the same value for chrysotile and mixed cohorts: "A single lung cancer exposure response model adequately describes the lung cancer data from Plants 1 and 2 combined" (p. 30). Hodgson and Darnton (2000) provided separate K_Ls for chrysotile-, amosite- and crocidolite-exposed cohorts.

[h] This 2nd K_L estimate was ignored (McDonald et al. 1983), so that only one K_L estimate per cohort (Hein et al. 2007) was used in the meta-analysis

[i] This 2nd K_L estimate was ignored (McDonald et al. 2004), so that only one K_L estimate per cohort (Sullivan 2007) was used in the meta-analysis

Supplemental Material, Table 4. Sensitivity analysis comparing results from the random effects meta-analysis in which studies were eliminated stepwise with specific exposure assessment descriptors, using potency estimates from the current study, Berman and Crump (2008a, 2008b) and Hodgson and Darnton (2000)

	Current study (n=19 studies)			B&C (2008a) (n=15 studies)			B&C (2008b) (n=18 studies)			H&D (2000) (n=21 studies)		
**Exclusion**	**Meta- K_L*100**	**SE**	**Studies included**	**Meta- K_L*100**	**SE**	**Studies included[a]**	**Meta- K_L*100**	**SE**	**Studies included[a]**	**Meta- R_L[b]**	**SE**	**Studies included[a]**
All studies	0.13	0.04	1-19	0.03	0.03	1-4, 6-8, 10-12, 12.2, 13, 16-18	0.06	0.03	1-4, 6-18	1.69	0.46	1-4, 4.1, 6, 7, 10-12, 12.1, 12.2, 13, 15-18, 20-23
- Studies with insufficient documentation	0.18	0.07	1, 4, 5, 8, 9, 12, 13, 15, 17, 18, 19	0.03	0.03	1, 4, 8, 12, 12.2, 13, 17, 18	0.11	0.05	1, 4, 8, 9, 12, 12.2, 13, 15, 17, 18	1.36	0.69	1, 4, 12, 12.1, 12.2, 13, 15, 17, 18
- studies with external conversion factors	0.19	0.08	1, 4, 5, 8, 9, 12, 13, 18, 19	0.03	0.03	1, 4, 8, 12, 12.2, 13, 18	0.12	0.07	1, 4, 8, 9, 12, 12.2, 13, 18	1.65	0.80	1, 4, 4.1, 12, 12.1, 12.2, 13, 18
- studies with insufficient job histories	0.36	0.12	4, 8, 9, 18, 19	0.62	0.59	1, 4, 8, 18	0.44	0.18	4, 8, 9, 18	3.32	1.25	4, 4.1, 18
- studies with CE ratio ≤50	0.56	0.21	4, 9, 19	1.80	1.47	4	0.57	0.35	4, 9	5.35	1.45	4, 4.1
- studies with coverage ≤30%	0.55	0.21	4, 9	1.80	1.47	4	0.57	0.35	4, 9	5.35	1.45	4, 4.1

n = number of unique populations/cohorts studied.
Exposure assessment covariates were not assessed for the studies which were included by B&C or H&D, but not included in the current study (no. 20-26; refer to Supplemental Material, Table 3). 18 of the 21 studies included by H&D overlapped with the studies included in the current analysis; exposure assessment covariates for non-overlapping studies (no. 23-26) were not classified. All studies included by B&C were classified.
[a] Refer to column 'No.' in Supplemental Material, Table 3 for study numbers
[b] R_L is the cohort average percentage excess of expected lung cancer mortality per unit of cumulative exposure

Supplemental Material, Table 5. Sensitivity analysis comparing results from the random effects meta-analysis in which studies were eliminated stepwise with specific exposure assessment descriptors: using K_L values calculated with the uppermost cumulative exposure category omitted, and the regression line forced through an intercept of 1

	Primary analysis			Upper CE category excluded			Fixed intercept (α = 1)			
**Exclusion**	**Meta-K_L*100**	**SE**	**AIC**	**Meta-K_L*100**	**SE**	**AIC**	**Meta-K_L*100**	**SE**	**AIC**	**Studies included**
All 19 studies	0.13	0.04	28.2	0.14	0.07	45.0	1.30	0.40	91.7	1-19
- Studies with insufficient documentation	0.18	0.07	30.6	0.16	0.12	46.7	1.01	0.54	89.5	1, 4, 5, 8, 9, 12, 13, 15, 17, 18, 19
- studies with external conversion factors	0.19	0.08	30.6	0.16	0.14	46.7	1.19	0.63	90.1	1, 4, 5, 8, 9, 12, 13, 18, 19
- studies with insufficient job histories	0.35	0.12	26.4	0.46	0.25	44.0	1.81	0.88	89.5	4, 8, 9, 18, 19
- studies with CE ratio ≤50	0.56	0.21	25.0	1.15	0.45	39.4	2.74	1.22	87.8	4, 9, 19
- studies with coverage ≤30%	0.55	0.21	25.3	1.13	0.45	39.6	1.74	1.24	89.2	4, 9

Supplemental Material, Table 6. Sensitivity analysis comparing random effects meta-analysis and subgroup analyses using potency estimates from the current study, Berman and Crump (2008b) and Hodgson and Darnton (2000)

	Current study			B&C (2008b)			H&D (2000)		
	**Meta-K_L*100**	**95% CI**	**p-value**	**Meta-K_L*100**	**95% CI**	**p-value**	**Meta-R_L[a]**	**95% CI**	**p-value**
All studies	0.13	0.04–0.22	-	0.06	0.00–0.12	-	1.69	0.80–2.59	-
Excluding Gustavsson et al. 2002	0.13	0.04–0.22	-	-	-	-	-	-	-
Documentation									
Insufficient	0.11	-0.04–0.26	0.46	0.05	-0.09–0.19	0.54	1.98	0.45–3.52	0.56
Sufficient	0.18	0.04–0.33		0.11	0.01–0.21		1.36	0.02–2.71	
Fiber									
Chrysotile	0.04	-0.05–0.12	0.06	0.04	-0.02–0.10	0.38	1.41	0.32–2.50	0.004
Amphiboles	0.33	0.09–0.56		0.38	-0.25–1.02		4.30	2.68–5.92	
Mixed	0.13	0.03–0.23		0.09	0.00–0.19		0.64	-0.17–1.44	
CE ratio (highest : lowest exposure category)									
≤50	0.10	-0.05–0.26	0.38	0.13	0.02–0.25	0.10	0.97	-0.46–2.40	0.25
>50	0.20	0.04–0.35		0.03	0.01–0.05		2.16	0.84–3.48	
Conversion factor (mppcf to f-yr/ml)									
External or never PCM	0.12	-0.07–0.30	0.69	0.12	0.00–0.25	0.17	1.95	0.28–3.61	0.65
Internal or always PCM	0.16	0.03–0.28		0.03	0.01–0.05		1.45	0.16–2.74	
Coverage of exposure data									
≤30%	0.08	-0.01–0.18	0.08	0.03	0.01–0.05	0.02	1.32	-0.08–2.71	.[b]
>30%	0.27	0.08–0.46		0.35	0.12–0.58		2.01	0.51–3.51	
Job histories									
Insufficient	0.03	-0.10–0.17	0.08	0.04	-0.04–0.13	0.41	1.20	-0.59–2.99	0.53
Sufficient	0.19	0.08–0.30		0.10	0.01–0.20		1.89	0.50–3.28	

Random effects meta-analysis models
[a] R_L is the cohort average percentage excess of expected lung cancer mortality per unit of cumulative exposure
[b] Mixed models did not converge and we could not get a precise p-value

Supplemental Material, Table 7. Sensitivity analysis comparing random effects meta-analysis and subgroup analyses using K_L values calculated with the uppermost cumulative exposure category omitted, and the regression line forced through an intercept of 1

	Primary analysis			Upper CE category excluded			Fixed intercept (α = 1)			Studies included[b]
	Meta-K_L*100	95% CI	p-value[a]	Meta-K_L*100	95% CI	p-value[a]	Meta-K_L	95% CI	p-value[a]	
All studies	0.13	0.04–0.22	-	0.14	-0.02–0.31	-	1.30	0.45–2.15	-	1-19
Excluding Gustavsson et al. 2002	0.13	0.04–0.22	-	0.14	-0.02–0.31	-	1.17	0.37–1.98	-	1-18
Documentation										
Insufficient	0.11	-0.04–0.26	0.46	0.20	-0.10–0.49	0.87	1.69	0.39–3.00	0.42	2, 3, 6, 7, 10, 11, 14, 16
Sufficient	0.18	0.04–0.33		0.16	-0.09–0.42		1.01	-0.14–2.16		1, 4, 5, 8, 9, 12, 13, 15, 17-19
Fiber										
Chrysotile	0.04	-0.05–0.12	0.06	0.10	-0.19–0.38	0.43	0.61	-0.73–1.96	0.08	1-5
Amphiboles	0.33	0.09–0.56		0.44	-0.06–0.94		2.86	1.27–4.46		6-9
Mixed	0.13	0.03–0.23		0.12	-0.11–0.34		0.90	-0.15–1.95		10-19
CE ratio (highest : lowest exposure category)										
≤50	0.10	-0.05–0.26	0.38	0.18	-0.11–0.47	0.97	0.90	-0.36–2.16	0.35	2, 3, 8, 11, 13, 15, 16, 17, 18
>50	0.20	0.04–0.35		0.17	-0.08–0.42		1.69	0.48–2.91		1, 4, 5, 6, 7, 9, 10, 12, 14, 19
Conversion factor (mppcf to f-yr/ml)										
External or never PCM	0.12	-0.07–0.30	0.69	0.23	-0.08–0.53	0.59	1.75	0.22–3.29	0.47	3, 7, 11, 15, 16, 17
Internal or always PCM	0.16	0.03–0.28		0.13	-0.10–0.35		1.10	0.04–2.16		1, 2, 4, 5, 6, 8-10, 12-14, 18, 19
Coverage of exposure data										
≤30%	0.08	-0.01–0.18	0.08	0.09	-0.08–0.26	0.24	1.40	0.26–2.54	0.82	4, 5, 9, 11, 12, 17, 18
>30%	0.27	0.08–0.46		0.32	-0.04–0.69		1.20	-0.23–2.64		1, 2, 3, 6, 7, 8, 10, 13, 14, 15, 16, 19
Job histories										
Insufficient	0.03	-0.10–0.17	0.08	0.01	-0.31–0.33	0.16	0.91	-0.67–2.48	0.44	1, 3, 5, 11, 12
Sufficient	0.19	0.08–0.30		0.30	0.04–0.55		1.50	0.44–2.55		2, 4, 6, 7, 8, 9, 10, 13-19

Random effects meta-analysis models
CE, cumulative exposure
[a] Statistical significance between subgroups
[b] Study numbers and references listed in Appendix

Supplemental Material, Figure 1. Funnel plot to assess potential publication bias

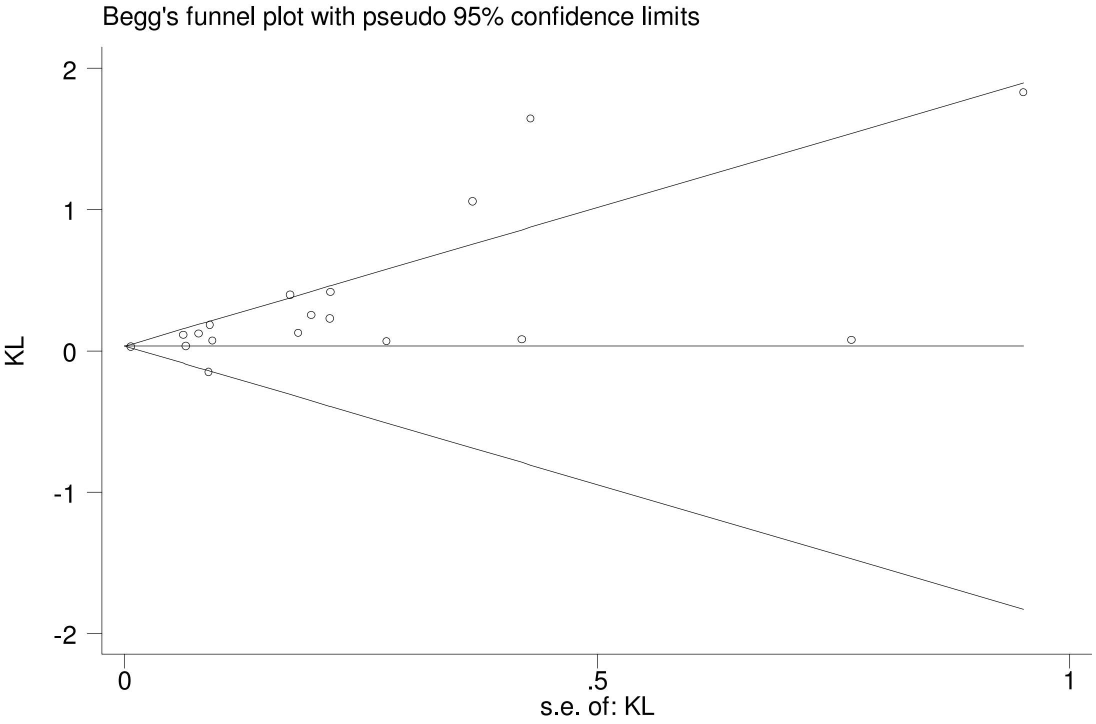

Note: study number 19 was excluded in constructing the plot to improve resolution

Begg adjusted rank correlation test (p = 0.17); Egger’s regression asymmetry test (p = 0.03)

Supplemental Material, Figure 2. Exclusion sensitiviy plot

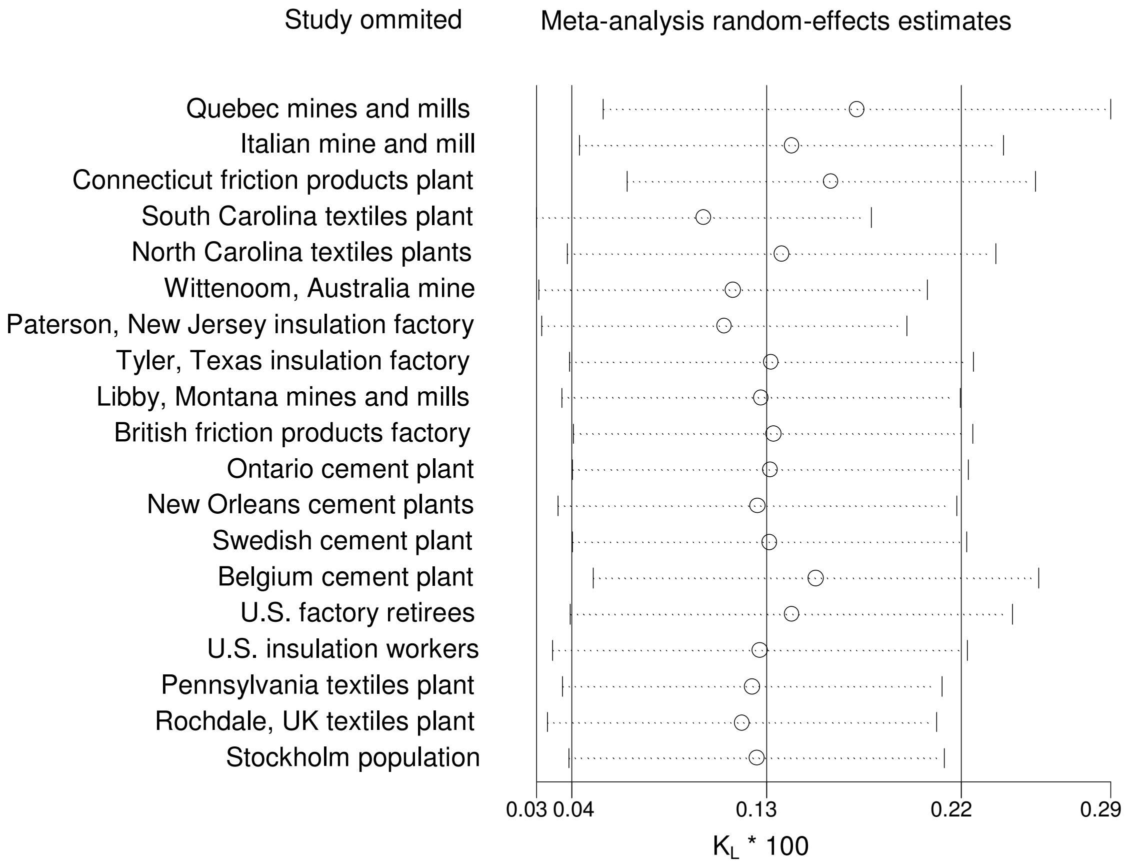

References

Albin M, Jakobsson K, Attewell R, Johansson L, Welinder H. 1990. Mortality and cancer morbidity in cohorts of asbestos cement workers and referents. Br J Ind Med 47:602-610.

Amandus HE, Wheeler R, Jankovic J, Tucker J. 1987. The morbidity and mortality of vermiculite miners and millers exposed to tremolite-actinolite: Part I. Exposure estimates. Am J Ind Med 11:1-14.

Berman DW, Crump KS. 2008a. A meta-analysis of asbestos-related cancer risk that addresses fiber size and mineral type. Crit Rev Toxicol 38(S1):49-73.

Berman DW, Crump KS. 2008b. Update of potency factors for asbestos-related lung cancer and mesothelioma. Crit Rev Toxicol 38(S1):1-47.

Berry G, Newhouse M. 1983. Mortality of workers manufacturing friction materials using asbestos. Br J Ind Med 40:1-7.

Berry G, de Klerk NH, Reid A, Ambrosini GL, Olsen NJ, Merler E, et al. 2004. Malignant and pleural mesothelioma in former miners and millers of crocidolite in Wittenoom, Western Australia. Occup Environ Med 61:1-3.

de Klerk NH, Armstrong BK, Musk AW, Hobbs MS. 1989. Cancer mortality in relation to measures of occupational exposure to crocidolite at Wittenoom Gorge in Western Australia. Br J Ind Med 46:529-536.

Dement JM, Harris RL Jr, Symons MJ, Shy CM. 1983a. Exposures and mortality among chrysotile asbestos workers. Part I: Exposure estimates. Am J Ind Med 4:399-419.

Dement JM, Harris, RL Jr, Symons MJ, Shy CM. 1983b. Exposures and mortality among chrysotile workers. Part II: Mortality. Am J Ind Med 4:421-433.

Dement JM, Myers D, Loomis D, Richardson D, Wolf S. 2009. Estimates of historical exposures by phase contrast and transmission electron microscopy in North Carolina USA asbestos textile plants. Occup Environ Med 66:574-583.

Enterline P, Hartley E, Henderson, J. 1987. Asbestos and cancer: a cohort followed up to death. Br J Ind Med 44:396-401.

Finkelstein M. 1984. Mortality among employees of an Ontario asbestos-cement factory. Am Rev Respir Dis 129:754-761.

Gustavsson P, Nyberg F, Pershagen G, Schéele P, Jakobsson R, Plato N. 2002. Low-dose exposure to asbestos and lung cancer: Dose-reponse relations and interaction with smoking in a population-based case-referent study in Stockholm, Sweden. Am J Epidemiol 155:1016-1022.

Hammad YY, Diem J, Weill H. 1979. Evaluation of dust exposure in asbestos cement manufacturing operations. Am Ind Hyg Assoc J 40:490-495.

Hein MJ, Stayner L, Lehman E, Dement JM. 2007. Follow-up study of chrysotile textile workers: Cohort mortality and exposure-response. Occup Environ Med 64:616-625.

Henderson VL, Enterline PE. 1979. Asbestos exposure: factors associated with excess cancer and respiratory disease mortality. Ann N Y Acad Sci 330:117-126.

Hodgson JT, Darnton A. 2000. The quantitative risks of mesothelioma and lung cancer in relation to asbestos exposure. Am Occup Hyg 44:565-601.

Hughes J, Weill H, Hammad Y. 1987. Mortality of workers employed in two asbestos cement manufacturing plants. Br J Ind Med 44:61-174.

Lacquet LM, van der Linden L, Lepoutre J. 1980. Roentgenographic Lung Changes, Asbestosis and Mortality in a Belgian Asbestos-Cement Factory. In: Biological Effects of Mineral Fibres, Wagner JC (ed.). IARC Sci Publ 783-793.

Levin JL, McLarty JW, Hurst GA, Smith AN, Frank AL. 1998. Tyler asbestos workers: mortality experience in a cohort exposed to amosite. Occup Environ Med 55:155-160.

Liddell F, McDonald A, McDonald J. 1997. The 1891- 1920 birth cohort of Quebec chrysotile miners and millers: development from 1904 and mortality to 1992. Ann Occup Hyg 41:13-36.

Loomis D, Dement JM, Wolf SH, Richardson DB. 2009. Lung cancer mortality and fibre exposures among North Carolina asbestos textile workers. Occup Environ Med 66:535-542.

McDonald AD, Fry JS, Wooley AJ, McDonald JC. 1983. Dust exposure and mortality in an American factory using chrysotile, amosite and crocidolite in mainly textile manufacturing. Br J Ind Med 40:368-374.

McDonald A, Fry J, Woolley A, McDonald J. 1984. Dust exposure and mortality in an American chrysotile asbestos friction products plant. Br J Ind Med 41:151-157.

McDonald JC, Harris J, Armstrong B. 2004. Mortality in a cohort of vermiculite miners exposed to fibrous amphibole in Libby, Montana. Occup Environ Med 614:363-366.

Neuberger M, Kundi M. 1990. Individual asbestos exposure: smoking and mortality—a cohort study into the asbestos cement industry. Br J Ind Med 47:615-620.

Nicholson WJ. 1976. Part III. Recent approaches to the control of carcinogenic exposures. Case study 1: Asbestos—The TLV approach. Ann NY Acad Sci 271:152-169.

Peto J, Doll R, Hermon C, Binns W, Clayton R, Goffe T. 1985. Relationship of mortality to measures of environmental asbestos pollution in an asbestos textile factory. Ann Occup Hyg 293:305-355.

Piolatto G, Negri E, La Veccia C, Pira E, Decarli A, Peto J. 1990. An update of cancer mortality among chrysotile asbestos miners in Balangero, Northern Italy. Br J Ind Med 47:810-814.

Pira E, Pelucchi C, Piolatto PG, Negri E, Bilei T, La Vecchia C. 2009. Mortality from cancer and other causes in the Balangero cohort of chrysotile asbestos miners. Occup Environ Med 66:805-9.

Rubino GF, Piolatto F, Newhouse ML, Scansetti G, Aresini GA, Murray R. 1979. Mortality of chrysotile asbestos workers at the Balangero Mine, Northern Italy. Br J Ind Med 36:187-194.

Seidman H, Selikoff IJ, Gelb SK. 1986. Mortality experience of amosite asbestos factory workers: dose-response relationships 5 to 40 years after onset of short-term work exposure. Am J Ind Med 10:479-514.

Seidman H, Selikoff IJ, Hammond EC. 1979. Short-term asbestos work exposure and long-term observation. Ann N Y Acad Sci 330:61-89.

Selikoff IJ, Hammond EC, Seidman H. 1979. Mortality experience of insulation workers in the United States and Canada 1943–1976. Ann NY Acad Sci 330:91-116.

Selikoff IJ, Seidman H. 1991. Asbestos-associated deaths among insulation workers in the United States and Canada, 1967–1987. Ann NY Acad Sci 643:1-14.

Sluis-Cremer GK, Liddell FDK, Logan WPD, Bezuidenhout BN. 1992. The mortality of amphibole miners in South Africa, 1946–80. Br J Ind Med 49:566-575.

Sullivan PA. 2007. Vermiculite, respiratory disease, and asbestos exposure in Libby, Montana: update of a cohort mortality study. Environ Health Perspect 115:579-585.

Talcott JA, Thurber WA, Kantor AF, Gaensler EA, Danahy JF, Antman KA, et al. 1989. Asbestos associated diseases in a cohort of cigarette-filter workers. N Engl J Med 321:1220-1223.

U.S. EPA. (U.S. Environmental Protection Agency). Airborne Asbestos Health Assessment Update. Washington, DC: U.S. EPA; 1986. EPA/6000/8-84/003E.
